# The Impact of Periodontitis on Oral Health Outcomes in Older Adults: A Systematic Review

**DOI:** 10.1111/ger.70048

**Published:** 2026-01-16

**Authors:** Lewis Winning, Kristina Bertl, Ioannis Polyzois, Yvonne Van der Waal, Rawan Kahatab, Peter Harrison, Gerry McKenna

**Affiliations:** ^1^ Dublin Dental University Hospital, Trinity College Dublin Ireland; ^2^ Department of Periodontology, Dental Clinic, Faculty of Medicine Sigmund Freud University Vienna Austria; ^3^ University of Groningen Groningen the Netherlands; ^4^ Centre of Public Health Queen's University of Belfast Institute of Clinical Sciences Belfast UK

**Keywords:** gerodontology, OHRQoL, periodontitis, systematic review, tooth loss

## Abstract

**Objective:**

To systematically review the impact of periodontitis on clinical oral health outcomes and oral health‐related quality of life (OHRQoL) in older adults (≥ 60 years).

**Background:**

Global evidence shows that severe periodontitis prevalence peaks in the 60s, yet how periodontitis relates specifically to oral health outcomes in older adults has not been comprehensively synthesised. Prior reviews in mixed‐age populations demonstrate strong links with tooth loss but less consistent associations with OHRQoL.

**Methods:**

This systematic review followed PRISMA guidelines and was registered on PROSPERO (CRD420251010568). Searches of PubMed, MEDLINE, Embase, Scopus, and Cochrane Library (to May 2025) identified studies of adults ≥ 60 years with periodontitis reporting oral health outcomes or OHRQoL. Two reviewers independently screened studies, extracted data, and assessed risk of bias.

**Results:**

Forty‐three studies met inclusion criteria: 18 longitudinal studies on clinical outcomes and 25 cross‐sectional studies on OHRQoL. Clinical studies consistently demonstrated that deeper baseline periodontal pockets, greater clinical attachment loss, and higher CPI/CPITN scores predicted adverse outcomes over 18 months to 12 years follow‐up, including tooth loss, further attachment loss, and functional decline. OHRQoL studies showed inconsistent findings, with roughly equal numbers reporting significant associations versus no relationship.

**Conclusions:**

In adults ≥ 60 years, periodontitis consistently predicts tooth loss and continued periodontal breakdown, whereas OHRQoL associations are inconsistent and appear mediated primarily through downstream consequences such as tooth loss and functional impairment, though direct effects may occur in advanced disease. These findings emphasise the importance of periodontal prevention and management in older adults.

## 1 Introduction

Periodontitis is a common chronic inflammatory disease that becomes increasingly prevalent with age and is a major contributor to the global oral disease burden [1]. Global estimates show severe periodontitis peaks at ages 60–64 years with strong age gradients across the 60s and beyond [2, 3]. This pattern is reflected in national data; for example in the United States, the National Health and Nutrition Examination Survey (NHANES) 2009–2012 estimated ~68% of adults aged ≥ 65 years had periodontitis, with ~11% severe periodontitis [4]. These patterns sit within a broader global picture in which oral conditions, including severe periodontitis, remain highly prevalent and unequally distributed across socioeconomic, geographic, and demographic lines [5].

The consequences of periodontitis extend well beyond the periodontium itself. Periodontal breakdown is a major driver of tooth loss in adults with periodontitis [6]. Tooth loss, in turn, creates a cascade of functional and quality‐of‐life impacts, being consistently linked to impaired chewing efficiency, altered diet and nutrition, and diminished oral health‐related quality of life (OHRQoL), with effects that vary according to the number, location, and distribution of missing teeth [7, 8]. Among older populations, dentition status encompasses the presence, distribution, and stability of remaining teeth and serves as a key determinant of oral function, with tooth loss emerging as the most commonly identified risk factor for masticatory dysfunction [9]. Beyond these oral impacts, studies also demonstrate significant systemic health consequences, with periodontitis associated with incident type 2 diabetes [10], reduced respiratory function [11], coronary heart disease [12], and all‐cause mortality [13]. These associations underscore that periodontal health represents a component of overall health rather than an isolated oral condition.

Clinical adoption of the 2017 World Workshop classification system, incorporating staging for severity and complexity alongside grading for rate and risk, offers a standardised framework to interpret disease trajectories and stratify periodontal risk [14]. However, synthesis of how periodontitis relates to key oral health outcomes in older adults remains fragmented. Studies examining the impact of periodontitis vary considerably in case definitions (using different combinations of probing pocket depth [PPD]), clinical attachment level (CAL), radiographic periodontal bone loss, or Community Periodontal Index/Community Periodontal Index of Treatment Needs (CPI/CPITN) measures, outcome assessments, follow‐up periods, and analytic approaches [15, 16, 17]. This heterogeneity hampers clinical understanding and evidence‐based decision making for older patients with periodontitis. Recent meta‐analyses confirm associations between periodontitis and poorer OHRQoL but highlight substantial between‐study heterogeneity and effect modification by comorbidities, underscoring the critical need for age‐specific evidence synthesis [18]. Given the distinct physiological, social and healthcare contexts of ageing, a focused systematic review examining the impact of periodontitis specifically in older adults is essential to inform clinical practice and guide targeted interventions.

The aim of this systematic review is therefore to examine the impact of periodontitis on clinical and functional outcomes (including tooth loss, attachment loss, periodontal disease progression, need for prosthetic rehabilitation, chewing efficiency, speech, and swallowing) and oral health‐related quality of life in adults aged ≥ 60 years.

## 2 Materials and Methods

This systematic review was conducted and reported in accordance with the Preferred Reporting Items for Systematic Reviews and Meta‐Analyses (PRISMA) guidelines [19, 20]. The protocol was prospectively registered on the International Prospective Register of Systematic Reviews (PROSPERO; registration number CRD420251010568). Focus questions, search strategy and selection procedures are outlined in Table 1, and summarised below.

**TABLE 1:** Search strategy and selection criteria.

Focus question	In older adults (≥ 60 years), how does periodontitis impact clinical oral health outcomes? (Including tooth loss, periodontal disease progression, and functional impairments.) In older adults (≥ 60 years), how does periodontitis impact oral health‐related quality of life?
Population	Older adults aged ≥ 60 years. Excluded if conducted exclusively in younger adults or exclusively in special populations with conditions substantially altering periodontal disease presentation/progression (e.g., poorly controlled diabetes, immunodeficiency). Mixed‐age studies eligible only if data for ≥ 60 years were reported separately
Exposure	Periodontitis, assessed using clinical parameters such as established case definitions, staging/grading, probing pocket depth (PPD), clinical attachment loss (CAL), bleeding on probing, radiographic bone loss, or the Community Periodontal Index (CPI)/Community Periodontal Index of Treatment Needs (CPITN). Studies without a clear definition or clinical assessment of periodontitis, or focusing solely on gingivitis/gingival inflammation, were excluded
Comparison	No specific comparator group required
Outcome	Clinical and functional outcomes—tooth loss, attachment loss, periodontal disease progression, need for prosthetic rehabilitation, chewing efficiency, speech, swallowing. Quality of life outcomes—oral health‐related quality of life (OHRQoL) measured using validated instruments such as GOHAI, OHIP, or OIDP. Studies were excluded if they did not report oral health impacts or if they focused exclusively on systemic outcomes without linking them to oral health
Language	No language restriction
Search date	Up to 8th May 2025
PROSPERO	CRD420251010568 (Registered 13 March 2025)
Database search	
Databases	PubMed, MEDLINE (Ovid), Embase, Scopus, and the Cochrane Library
Supplementary handsearch	
Journals (March 2020 to March 2025)	*Gerodontology*. *Journal of Periodontology*. *Journal of Clinical Periodontology*. *Journal of Periodontal Research*
References	Included articles and identified reviews
Grey literature search	
Conference proceedings	Europerio 2018, 2022 Abstracts
Study registers	ANZCTR, ChiCTR, EU‐CTR, DRKS, ISRCTN, ClinicalTrials.gov
Selection process	
Inclusion criteria	Population—Older adults (≥ 60 years). Exposure—periodontitis defined clinically (case definition, stage/grade, PPD, CAL, radiographic bone loss, CPI/CPITN). Outcomes—clinical outcomes: tooth loss, attachment loss, progression of periodontal disease, need for prosthetic rehabilitation; Functional outcomes: chewing efficiency, speech, swallowing; Quality of life outcomes: OHRQoL (GOHAI, OHIP, or OIDP). Study design—observational cohort studies (prospective/retrospective), cross‐sectional studies, case–control studies; interventional studies (randomised/non‐randomised) where the intervention does not modify the natural association between periodontitis and oral health outcomes
Contact with authors	Applied when studies potentially met inclusion criteria but full text was unavailable, data were incomplete or unclear, or additional information was required from grey literature sources
Exclusion criteria	Population—exclusively < 60 years; special populations as above. Exposure—no clear definition or assessment of periodontitis; gingivitis‐only studies. Outcomes—no relevant oral health outcomes; systemic‐only outcomes without oral health linkage. Study design—for Focus Question 1 (clinical oral health outcomes), designs other than longitudinal cohort studies were excluded; for Focus Question 2 (OHRQoL outcomes), designs other than observational studies (including cross‐sectional) were excluded. Case reports, narrative reviews, letters, editorials, conference abstracts; animal or in vitro studies; studies where the intervention specifically targeted periodontal treatment, altering its natural effect on outcomes
Identification process	Titles/abstracts saved in Covidence following duplicate removal. Two reviewers screened independently; disagreements resolved by discussion with a third reviewer. Cohen's *κ* calculated for inter‐rater agreement at both title/abstract and full‐text stages

### 2.1 Focus Questions

Two focus questions were addressed:

- In older adults (≥ 60 years), how does periodontitis impact clinical oral health outcomes? This included tooth loss, periodontal disease progression, and functional impairments.
- In older adults (≥ 60 years), how does periodontitis impact oral health‐related quality of life?

### 2.2 Eligibility Criteria

#### 2.2.1 Population

Older adults aged ≥ 60 years. The ≥ 60‐year threshold was selected as it aligns with global epidemiological reporting of periodontitis burden and ageing populations, including Global Burden of Disease analyses, and is commonly used in gerodontological and periodontal research to capture the age range in which severe periodontitis prevalence peaks [2, 3, 5]. Studies exclusively involving younger adults, or exclusively conducted in special populations with conditions that substantially modify periodontal disease presentation or progression (e.g., poorly controlled diabetes, immunodeficiency, or other systemic conditions requiring specialised management), were excluded. Mixed‐age studies were eligible only if data were reported separately for participants aged ≥ 60 years.

#### 2.2.2 Exposure

Periodontitis, assessed using clinical parameters such as established case definitions, staging/grading, PPDs, CAL, radiographic bone loss, or CPI/CPITN measures. Studies without a clear definition or clinical assessment of periodontitis, or focusing solely on gingivitis and gingival inflammation were excluded.

#### 2.2.3 Comparator

No specific comparator group was required.

#### 2.2.4 Outcomes

- Clinical and functional outcomes—tooth loss, attachment loss, periodontal disease progression, need for prosthetic rehabilitation, chewing efficiency, speech, and swallowing.
- Quality of life outcomes—OHRQoL, measured using validated instruments such as the Geriatric Oral Health Assessment Index (GOHAI), Oral Health Impact Profile (OHIP), or Oral Impacts on Daily Performance (OIDP).

Studies were excluded if they did not report oral health impacts or if they focused exclusively on systemic outcomes without linking them to oral health (Table 1).

#### 2.2.5 Study Characteristics

For clinical outcomes, eligible designs included longitudinal observational studies (prospective or retrospective cohorts) and interventional studies (randomised or non‐randomised) where the intervention did not alter the natural association between periodontitis and oral health outcomes. For OHRQoL outcomes, observational studies including cross‐sectional designs were also eligible. Case reports, narrative reviews, letters, editorials, conference abstracts, animal or in vitro studies, and interventional studies targeting periodontal treatment (e.g., surgical or supportive therapy) were excluded.

### 2.3 Information Sources and Search Strategy

A single comprehensive search strategy was developed to address both review questions, covering periodontitis and its impact on clinical oral health outcomes and OHRQoL in older adults. Searches were conducted in PubMed, MEDLINE (Ovid), Embase, Scopus, and the Cochrane Library. The databases were searched from their inception (earliest available records) to 8 May 2025. The strategy combined controlled vocabulary (e.g., MeSH and Emtree terms) and free‐text keywords related to:

- Population—older adults (e.g., ‘Older Adults’, ‘Geriatric Population’, ‘Aged 60’, ‘Senior*’, ‘Gerodont*’).
- Exposure—periodontitis (e.g., ‘Periodontitis’ [MeSH], ‘periodont*’, ‘Periodontal Index’, ‘Periodontal Atrophy’).
- Outcomes—oral health outcomes including tooth loss, attachment loss, disease progression, edentulism, tooth extraction, functional impairments (mastication, speech, swallowing), and OHRQoL (e.g., ‘GOHAI’, ‘OHIP’, ‘OIDP’, ‘oral health‐related quality of life’).

Boolean operators (OR, AND) and proximity operators (e.g., NEAR/2, adj2) were used to combine terms within and across these domains. The search strategy was refined iteratively to improve specificity, with some terms removed (e.g., ‘Gingival Index’, ‘Periodontal Status’) and others added (e.g., ‘Periodontal Atrophy’, ‘Tooth Extraction’). Database‐specific syntax and subject headings were adapted for each platform. Reference lists of all included studies and relevant reviews were also hand‐searched to identify additional eligible studies. The full search terms for each database are provided in Data S1.

### 2.4 Study Selection

Search results were imported into Covidence (Himmelfarb Health Sciences Library, Washington DC, USA), where duplicates were removed automatically and then verified manually before screening. Two reviewers independently screened titles and abstracts against the eligibility criteria. Where eligibility could not be determined from the abstract, the full text was retrieved and assessed. Disagreements were resolved by discussion with a third reviewer. Inter‐rater agreement was calculated using Cohen's *κ*.

### 2.5 Data Extraction

A standardised data extraction form was developed and piloted. Extracted data included:

- Study characteristics: author, year, country, design, sample size
- Population: age, gender, setting, relevant health status
- Exposure: periodontal case definition and measures (e.g., staging/grading, PPD, CAL, bleeding on probing, radiographic bone loss, CPI/CPITN)
- Outcomes: type of oral health outcome, measurement tools, and key results (effect estimates, *p*‐values, confidence intervals)

Data were extracted independently by two reviewers, with discrepancies resolved by consensus.

### 2.6 Risk of Bias Assessment

The Newcastle–Ottawa Scale (NOS) was used to assess the methodological quality of observational studies, adapted for cross‐sectional and cohort designs as appropriate. Domains assessed included selection, comparability, and outcome assessment. Where reporting was unclear, no star was awarded, in line with established NOS guidance [21].

### 2.7 Data Synthesis

Due to anticipated heterogeneity in study populations, definitions of periodontitis, and outcome measures, a narrative synthesis was performed. Meta‐analysis was not undertaken because of substantial variation across studies in periodontal case definitions (PPD, CAL, CPI/CPITN and differing thresholds), tooth loss definitions, OHRQoL instruments (OHIP, GOHAI, OIDP), follow‐up durations, and analytic levels (tooth‐, person‐ and multi‐level analyses). Pooling such diverse measures would not have yielded clinically meaningful or methodologically robust estimates. Studies were therefore grouped by outcome domain (clinical/functional vs. OHRQoL) and summarised according to periodontal disease severity and measurement approach.

## 3 Results

### 3.1 Study Selection

**FIGURE 1:**
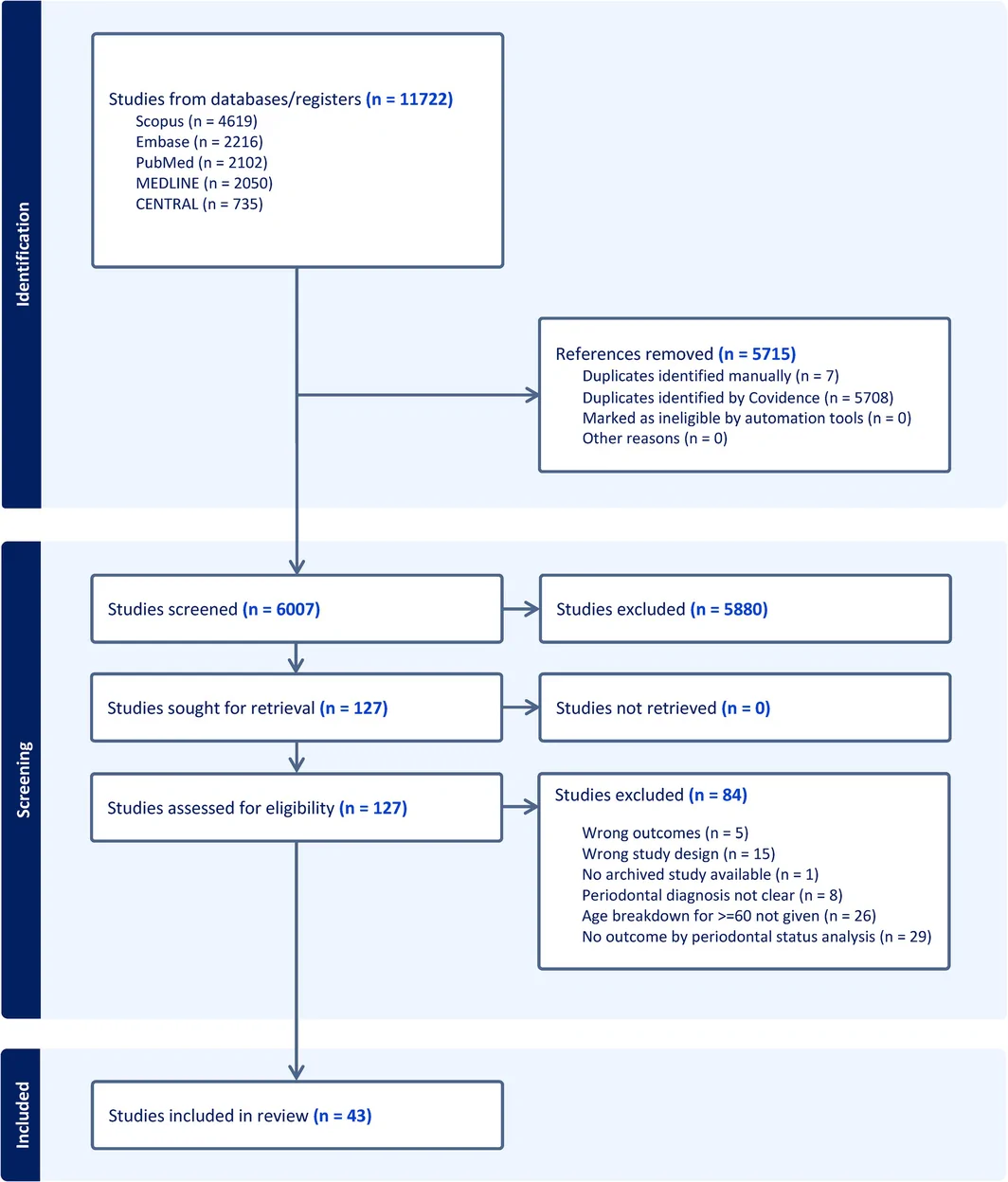
PRISMA flow diagram summarising the study selection process. [Colour figure can be viewed at wileyonlinelibrary.com]

The study selection process is summarised in Figure 1 (PRISMA flow diagram). A total of 11,722 records were identified through database searches: PubMed (*n* = 2101), MEDLINE (*n* = 2050), Embase (*n* = 2216), Scopus (*n* = 4619), and the Cochrane Library (*n* = 736; reviews and trials). After automated de‐duplication in Covidence (*n* = 5708) and manual removal (*n* = 7), 6007 unique records remained for title/abstract screening. Of these, 5880 were excluded for not meeting the inclusion criteria. The full texts of 127 articles were retrieved for detailed assessment, resulting in the exclusion of 84 studies. Reasons for exclusion were: absence of age breakdown for participants aged ≥ 60 years (*n* = 26); ineligible study design (*n* = 15); no outcome analysis by periodontal status (*n* = 29); unclear periodontal diagnosis (*n* = 8); wrong outcomes (*n* = 5); and unavailable archived study (*n* = 1). Full details of excluded studies and reasons are provided in Data S2.

In total, 43 studies met the inclusion criteria and were included in the qualitative synthesis: 18 studies addressed clinical oral health outcomes (Focus Question 1), and 25 studies addressed oral health‐related quality of life (Focus Question 2). Inter‐rater agreement across reviewers yielded a pooled Cohen's *κ* = 0.74, indicating moderate‐to‐substantial agreement [22].

### 3.2 Focus Question 1: Clinical Outcomes

#### 3.2.1 Study Characteristics

Table 2 presents the findings of the narrative synthesis specific to clinical outcomes. Eighteen cohort studies were included, all assessing the impact of baseline periodontal status on subsequent oral health outcomes in older adults. Studies were conducted in Australia (*n* = 1), Canada (*n* = 1), Germany (*n* = 1), Japan (*n* = 11), Sweden (*n* = 1), and the USA (*n* = 3). The number of participants completing follow‐up ranged from 39 to 3409, totalling over 13,000 older adults. Follow‐up durations ranged from 18 months to 12 years. No interventional studies met the inclusion criteria for this question.

**TABLE 2:** Study description: oral health outcomes.

Author	Country	Design	Study duration	Participants	Age criteria	Setting	Periodontal diagnosis	Outcome	Analysis	Outcome	Conclusion
Beck et al. [23]	USA	Cohort study	3 years (with assessments at 18 and 36 months)	*N* = 338	≥ 65 years	Community dwelling	Clinical attachment level (CAL) measured at buccal and mesio‐buccal sites	Periodontal breakdown defined as ≥ 3 mm CAL loss over an 18‐month interval	Weighted descriptive analysis	Baseline probing depth > 3 mm increased the risk of CAL loss (Blacks: 13.2% of sites with > 3 mm PPD lost attachment vs. 4.7% of shallow sites at 36 months; Whites: 7.4% vs. 2.4%). The majority of sites that did break down had probing depths ≤ 3 mm (80% of affected sites in Blacks; 95% in Whites)	Deeper baseline pockets were associated with greater risk of breakdown, but most events of progression occurred in shallow sites, limiting the predictive value of baseline CAL and PPD
Beck et al. [24]	USA	Cohort study	5 years	*N* = 396	≥ 65 years	Community dwelling	Worst level of attachment loss (≤ 3 mm; 4‐5 mm; ≥ 6 mm); and probing depth (> 3 mm) at baseline	Tooth loss (any tooth loss)	Multivariable logistic regression	Logistic regression analysis showed that the worst level of attachment per tooth at baseline OR = 1.88 (95% CI 1.39–2.55) was significantly associated with tooth loss over 5 years. Baseline pocket depth > 3 mm was also associated with tooth loss OR = 1.71 (95% CI 1.07–2.72)	Teeth with the poorest attachment level at baseline, and teeth that showed progressive attachment loss during the observation period were most likely to be lost over the 5 years
Beck et al. [25]	USA	Cohort study	5 years (with assessments at 18 months, 36 months, and 5 years)	*N* = 220	≥ 65 years	Community dwelling	Clinical attachment level (CAL) measured at buccal and mesio‐buccal sites	Patterns and rates of attachment loss in teeth that survived across the follow‐up	Longitudinal analysis of changes in attachment level by site and tooth; patterns assessed by descriptive statistics and incidence rate calculations; subgroup comparisons by baseline status	Greatest attachment loss occurred at sites with pre‐existing disease at baseline; molars—especially maxillary molars—were more affected; buccal and mesial sites had higher progression rates than lingual or distal sites	Surviving teeth continue to experience attachment loss, particularly those with baseline periodontal involvement
Brown et al. [26]	USA	Cohort study	18 months	*N* = 492	≥ 65 years	Community dwelling	Baseline ‘serious periodontitis’ (≥ 4 sites with ≥ 5 mm CAL and ≥ 1 site with ≥ 4 mm PPD)	Periodontal disease progression (≥ 2 sites with CAL increase ≥ 3 mm)	Bivariate logistic regression (by race)	Baseline serious periodontitis predicted further attachment loss: Blacks OR = 2.5 (95% CI 1.42–4.26); Whites OR = 6.0 (95% CI 2.66–13.78). Baseline periodontal status was not included in a multivariable logistic regression analysis	Baseline serious periodontitis increased the risk of further attachment loss in both groups
Gonda et al. [27]	Canada	Cohort study	5 years	*N* = 193	60–75 years of age (Mean 67 ± 4 years)	Community dwelling	No. of teeth with attachment loss ≥ 6 mm (0 or 1/2 or more)	Multiple Tooth loss (≥ 3 teeth)	Multivariable logistic regression	Logistic regression analysis revealed attachment loss ≥ 6 mm was significantly associated with ≥ 3 tooth loss during the 5 year observation period OR = 1.25 (95% CI 1.11–1.42)	Attachment loss was a significant predictor of multiple tooth loss after 5 years
Hassel et al. [28]	Germany	Cohort study	Up to 10 years	*N* = 39	Subgroup of 71–75 year olds (mean 73.8 ± 1.0 years)	Community dwelling	Deepest PPD (≤ 3/> 3 mm)	Tooth loss (per tooth)	Multivariable logistic regression	Tooth‐level logistic regression showed that PPD > 3 mm (OR = 2.4; 95% CI 1.6–3.4), at baseline was significantly associated with tooth loss	Baseline deep periodontal pockets were associated with subsequent toothloss
Hirotomi et al. [29]	Japan	Cohort study	2 years	*N* = 394	70 year olds	Community dwelling	Highest PPD and the highest CAL in a subject/tooth were defined as the representative values for that subject/tooth	Periodontal disease progression. CAL change of ≥ 3 mm. Tooth loss (per tooth)	Subject level and tooth level multivariate logistic regression	Tooth level logistic regression showed that teeth with 7+ mm PPD (OR = 1.80 95% CI 1.09–2.99) and teeth with 7+ mm CAL (OR = 1.32 95% CI 1.03–1.67), were significantly associated with further attachment loss. Tooth level logistic regression showed that 7 mm+ CAL (OR = 4.94 95% CI 3.51–6.95) was significantly associated with tooth loss, whereas 7 mm+ PPD was not (OR = 1.54 95% CI 0.86–2.75)	Baseline attachment loss was apredictor of both further attachment loss and tooth loss
Hirotomi et al. [30]	Japan	Cohort study	10 years (including a 5 year visit)	*N* = 286	70 year olds	Community dwelling	Deepest PPD/tooth (< 4/4–5/≥ 6 mm), and highest CAL/tooth (< 4/4–5/≥ 6 mm)	Periodontal disease progression defined as CAL ≥ 3 mm	Descriptive analysis	Teeth with the deepest baseline PPD ≥ 6 mm had the highest rates of further periodontal disease progression—43.3% over 5 years and 36.0% over 10 years. Similarly, teeth with the highest baseline CAL ≥ 6 mm had the highest rates of further periodontal disease progression—33.4% over 5 years and 28.6% over 10 years. Baseline periodontal status was not included in a multilevel logistic regression analysis	Teeth with deep PPD or severe CAL at baseline tended to exhibit periodontal disease progression
Hirotomi et al. [31]	Japan	Cohort study	10 years	*N* = 276 at 10 years	≥ 70 years	Community dwelling	Deepest PPD/tooth (< 4/4–6/≥ 6 mm), and highest CAL/tooth (< 4/4–5/≥ 6 mm)	Tooth loss	Multilevel logistic regression	Multilevel logistic regression identified baseline CAL (at a tooth level) as a significant predictor of toothloss over 10 years: CAL < 4 mm: (Ref) 4 ≤ CAL < 6 mm: OR = 2.02 (95% CI 1.56–2.62) CAL ≥ 6 mm: OR = 7.07 (95% CI 5.15–9.72)	Clinical attachment loss was a significant predictor of tooth loss over a 10 year observation period
Iwai et al. [32]	Japan	Cohort study	2 years	*N* = 3409	≥ 75 years (Mean = 81 years)	Community dwelling	CPI. PPD ≥ 4 mm. Absence: < 4. Presence: ≥ 4	Decline in swallowing function	Multivariate logistic regression	Logistic regression analysis revealed periodontal pocket depth ≥ 4 mm was significantly associated with a decline in swallowing function at 2 year follow‐up, OR: 1.47 (95% CI 1.16–1.86)	Periodontal pocket depth was associated with a decline in swallowing function after 2 years
Mihara et al. [33]	Japan	Cohort study	6 years	*N* = 528 (*n* = 296 70‐year‐old group, and *n* = 232 80‐year‐old group)	69–71 and 79–81‐year‐olds	Community dwelling	Mean PPD of all teeth	Tooth loss: Group 1 (0 teeth lost) Group 2 (4 or more teeth lost)	Multivariable logistic regression	Logistic regression analysis revealed that mean probing pocket depth (OR = 5.70, 95% CI 2.70–12.04) was significantly associated with the loss of four or more teeth	Mean periodontal pocket probing depth was a significant predictor for tooth loss over a 6 year observation period
Nilsson et al. [34]	Sweden	Cohort study	12 years	*N* = 375	Age cohorts: 60, 66, 72 and 78	Community dwelling	Periodontitis defined as ≥ 2 sites with ≥ 5 mm distance from CEJ to the marginal bone level and ≥ 1 tooth with pockets ≥ 5 mm	Tooth loss (≥ 3 teeth)	Multivariate logistic regression	Logistic regression analysis revealed that periodontitis (≥ 2 sites 5 mm and ≥ 1 tooth ≥ 5 mm pocket) was significantly associated with ≥ 3 tooth after 12 years OR = 2.9 (95% CI 1.8–4.9)	Periodontitis was a significant predictor for tooth loss after 12 years
Nishimoto et al. [35]	Japan	Cohort study	6 years	*N* = 1234	≥ 65 years (Mean 72.2 ± 5.1 years old)	Community dwelling	CPI. Highest score per participant. Moderate periodontitis (CPI 3). Severe periodontitis (CPI 4)	‘New‐onset oral frailty’ based on six criteria including tooth loss	Cox proportional hazards modelling	Baseline CPI 3 had no significant difference in the risk of oral frailty however HR = 1.09 (95% CI 0.83–1.44). Baseline CPI 4 was significantly associated with an increased risk of new‐onset oral frailty HR = 1.42 (95% CI 1.12–1.81)	Severe periodontitis (CPI 4) was associated with new‐onset oral frailty in this study
Ogawa et al. [36]	Japan	Cohort study	2 years	*N* = 394	70 year olds	Community dwelling	Periodontal attachment level 0: < 6 mm at most severe site. 1: ≥ 6 mm at most severe site	Periodontal disease progression, ≥ 3 mm increase in probing attachment level at one or more sites	Multivariable logistic regression	Logistic regression analysis showed that baseline attachment ≥ 6 mm was associated with disease progression OR = 2.3 (95% CI 1.4–3.8)	Baseline attachment level of: ≥ 6 mm may be considered a risk factor for further attachment loss
Sato et al. [37]	Japan	Cohort study	6 years	*N* = 812	≥ 70 years	Community dwelling	Deepest periodontal pocket depth (≤ 3, 4–5, ≥ 6 mm)	Tooth loss (any)	Bivariate GEE logistic regression (individual‐ and tooth‐level factors)	Baseline periodontal pocket was significantly associated with tooth loss. Bivariate analysis: ≤ 3 mm: (Ref); 4–5 mm: OR 1.83 (1.51–2.21) < 0.001; 6 mm: OR 6.98 (5.38–9.04); Multivariable: N/A	Deeper baseline pockets were strongly associated with higher odds of tooth loss over 6 years
Shimazaki et al. [38]	Japan	Cohort study	6 years	*N* = 418	Mean age 78.2 ± 7.5 years	Institutionalised elderlies	Deepest periodontal pocket depth on index teeth (≤ 3, 4–5, ≥ 6 mm)	Tooth loss (per tooth lost) and incidence of edentulism	Multiple logistic regression	Baseline periodontal pocket depth ≥ 4 mm had a *B* = 1.4 (*p* = 0.0002) per tooth lost. Baseline periodontal pocket depth ≥ 4 mm had an OR = 3.1 (95% CI 1.1–8.5) for 6‐year incidence of edentulism	Baseline deep periodontal pockets were strongly associated with both tooth loss and risk of becoming edentulous
Slade et al. [39]	Australia	Cohort study	2 years	*N* = 693	≥ 60 years (Mean 72.4 ± 0.52)	Community dwelling	Number of pockets 4+ mm, and number of sites with recession of 4+ mm	Tooth loss (At least 1 tooth lost)	Multivariable logistic regression	Multivariable logistic regression identified pockets 4+ mm (OR = 1.82 95% CI 1.28–2.58) and recession 4+ mm (OR = 1.61 95% CI 1.24–2.08)	Periodontal pockets and gingival recession were both a significant predictor for tooth loss
Warren et al. [40]	USA	Cohort study	Mean = 13.9 years (13–15 years)	*N* = 73	≥ 65 years	Community dwelling	Mean attachment loss (mm)	Tooth loss	Multivariable logistic regression	Multivariable logistic regression identified greater severity of attachment loss (per mm increase) as a significant risk factor for tooth loss (OR = 2.4, 95% CI 1.27–4.36)	Baseline attachment loss was a significant predictor for future tooth loss

Abbreviations: CAL, clinical attachment level; CPI, Community Periodontal Index; CPITN, Community Periodontal Index of Treatment Needs; HR, hazard ratio; OR, odds ratio; PPD, probing pocket depth.

All studies recruited participants aged ≥ 60 years; some restricted recruitment to fixed age groups (e.g., 70 years only or cohorts aged 71–75 years), while others included broader age ranges (mean ages 67–81 years). Sixteen studies (80%) involved community‐dwelling participants, while four involved institutionalised or mixed populations.

Baseline periodontal diagnosis was most frequently based on PPD (*n* = 12), followed by CAL or loss of attachment (*n* = 10), CPI/CPITN (*n* = 2), and radiographic bone loss (*n* = 1). Diagnostic thresholds varied, including PPD categories such as ≤ 3, 4–5, and ≥ 6 mm; loss of attachment categories such as < 4, 4–5, and ≥ 6 mm; and CPI/CPITN codes ranging from 0 to 4. Counts are not mutually exclusive.

#### 3.2.2 Periodontal Disease Progression

Four studies [23, 26, 29, 36] evaluated periodontal disease progression defined by clinical attachment loss of ≥ 3 mm, or related changes. Across these, deeper baseline pockets and greater loss of attachment consistently predicted further breakdown, though progression also occurred at shallow sites. Some studies used descriptive analyses [29] while three applied logistic regression at tooth or subject level [23, 26, 36].

#### 3.2.3 Tooth Loss

Ten studies [24, 27, 28, 31, 33, 34, 37, 38, 39, 40] assessed tooth loss as the primary outcome. Follow‐up periods ranged from 2 to 12 years. Multivariable analyses demonstrated that deeper baseline pockets, greater loss of attachment, and radiographic bone loss were significantly associated with tooth loss. Odds ratios for severe baseline loss of attachment predicting tooth loss ranged from 2.02 to 7.07. Definitions (≥ 1, ≥ 3, ≥ 4 teeth) and analysis levels varied (person‐, tooth‐, multi‐level) [24, 27, 31, 33, 34, 39]. Baseline CAL generally outperformed PPD as a predictor and showed dose–response [24, 29, 31]. Institutionalised cohorts seemed to have had potentially higher risks for tooth loss including incident edentulism [38].

#### 3.2.4 Periodontal Disease Progression and Tooth Loss Combined

Two studies [24, 29] reported on both periodontal breakdown and tooth loss. Both found that baseline loss of attachment was a stronger predictor of tooth loss than PPD, while deeper pockets better predicted further attachment loss.

#### 3.2.5 Other Oral Health Clinical Outcomes

Two studies examined outcomes beyond tooth loss or disease progression. Iwai and colleagues [32] found that PPD ≥ 4 mm at baseline was significantly associated with a decline in swallowing function after 2 years (OR 1.47, 95% CI 1.16–1.86). Nishimoto and co‐workers [35] reported that severe periodontitis (CPI code 4) was significantly associated with an increased risk of new‐onset oral frailty over 6 years (HR 1.42, 95% CI 1.12–1.81).

#### 3.2.6 Summary of Clinical Oral Health Outcomes

Across all outcomes, deeper baseline pockets, greater loss of attachment, and more severe CPI/CPITN scores consistently predicted poorer clinical oral health outcomes over follow‐up, including greater risk of tooth loss, further periodontal breakdown, or deterioration in functional outcomes. The strength of association was evident across a range of study designs and populations, from community‐dwelling older adults to institutionalised cohorts, and persisted over follow‐up periods extending up to 12 years. While most studies supported a dose–response relationship between baseline severity and adverse outcomes, one study reported that a substantial proportion of progression occurred at sites with shallow baseline probing depths [23], indicating that disease activity in older adults can also arise in less severely affected sites. This suggests that reliance on baseline severity alone may underestimate future risk and reinforces the need for ongoing monitoring irrespective of initial disease stage.

### 3.3 Focus Question 2: OHRQoL

#### 3.3.1 Study Characteristics

Table 3 presents the findings of the narrative synthesis specific to OHRQoL outcomes. Twenty‐five cross‐sectional studies assessed the association between clinical measures of periodontal status and OHRQoL in older adults. Studies were conducted across diverse geographic settings, including Asia (*n* = 15), Europe (*n* = 5), North America (*n* = 3), and South America (*n* = 2). Sample sizes ranged from 53 [43] to 4332 [65] participants, with the majority (*n* = 21) recruiting community‐dwelling older adults and four sampling institutionalised populations. Recruitment age criteria ranged from broad ranges such as 60–87 years [43] and 65–97 years [60] to narrower bands including 60–74 years [51], 60–80 years [64], and 65–74 years [61, 65]. Two studies sampled fixed cohorts aged ≥ 70 years [47, 52]. The oldest participant across all studies was 97 years.

**TABLE 3:** Study description: OHRQoL outcomes.

Author	Country	Design	Participants	Age criteria	Setting	Periodontal diagnosis	OHRQoL instrument	Outcome
Agustina et al. [41]	Indonesia	Cross‐sectional	*N* = 153 (39 males, 114 females)	≥ 60 years	Community elders	CPI (Score of 3 & 4), Simplified Oral Hygiene Index (OHI‐S)	GOHAI	Periodontal pocket depth was not associated with OHRQoL in bivariate (*r* = 0.846, *p* = 0.60) and multivariate analysis
Botelho et al. [42]	Portugal	Cross‐sectional	*N* = 592 (272 males, 320 females)	≥ 65 years	Community elders	According to AAP/EFP 2017 Workshop definition; localised vs. generalised and mild, moderate, severe (stage 1–3); also included: PPD, CAL, REC, BOP, missing teeth	OHIP‐14	The number of missing teeth, mean probing depth and mean clinical attachment loss were significantly associated with a frequently affected OHRQoL. The periodontal status itself did not influence the OHRQoL, rather the clinical features did
Guevara‐Canales et al. [43]	Peru	Comparative and cross‐sectional	*N* = 53 (no female/male numbers reported)	60–87 years	Adults who attended an oral health campaign at the Health Care Centre ‘Juan Pablo II’ and all the older adult patients who live at the home for the elders ‘Virgen del Amor Hermoso’ in Lima, Peru	CPI O'Leary's Hygiene index	OIDP (Oral Impacts on Daily Performance)	In older adults, the correlation between OIDP and CPI or Hygiene index was not statistically significant (*p* > 0.05)
Göktürk & Uçan Yarkaç [44]	Turkey	Cross‐sectional	*N* = 155 (71 males, 84 females)	65–97 years	Adults attending a university periodontal department	Gingival index, plaque index, probing pocket depth, clinical attachment level and bleeding on probing. Gingivitis and chronic periodontitis (mild vs. severe) were diagnosed in accordance with the 1999 International World Workshop	GOHAI OHIP‐14	Only 22 participants (14.2%) had a GOHAI score of 0, indicating no impact from periodontal conditions, and only 2 participants (1.3%) had an OHIP‐14 score of 0. Over two‐thirds of participants (70.96%) reported that their self‐rated oral health was poor. Bleeding gums were significantly associated with OHIP‐14. Mobile teeth and oral malodor were significantly associated with GOHAI. OHIP‐14 scores were related to the plaque index, and GOHAI was associated with gingival recession
Marya et al. [45]	India	Cross‐sectional	*N* = 1200 (746 males, 454 females)	≥ 60 years	Community‐based household survey and clinical examination	BOP, PPD, loss of attachment, tooth mobility	GOHAI‐12	There was a significant association between mobile teeth and OHRQoL (*p* = 0.0001). For gingival bleeding, periodontal pockets, and loss of attachment, there was no significant association (*p* > 0.05)
León et al. [46]	Chile	Validation study (cross‐sectional, discriminative cross‐validation)	*N* = 85 (33 males, 52 females)	≥ 60 years	Dental clinics of University of Talca, private practices, senior social clubs, primary healthcare centres	CPITN (simple or complex periodontal treatment), tooth number	OHIP‐49	Participants needing complex periodontal treatment had significantly higher OHIP‐49 scores across all dimensions (*p* < 0.0001 for each) compared to those needing only simple treatment
Kato et al. [47]	Sweden	Cross‐sectional	*N* = 804 (235 males, 569 females)	≥ 70 years	Community dwelling older adults	Localised vs. generalised, PPD, tooth number	OHIP‐14	Periodontitis did not show an association with poor OHRQoL, however, a significant association between the number of teeth and poor OHRQoL was found
Rekhi et al. [48]	India	Cross‐sectional	*N* = 500 (221 males, 279 females)	≥ 60 years	Care homes	CPI, Loss of attachment, tooth mobility	GOHAI	Greater loss of attachment, and tooth mobility were significantly associated with poorer OHRQoL. However and conversely, a higher CPI score was associated with a better OHRQoL score
Castrejón‐Pérez et al. [49]	Mexico	Cross‐sectional	*N* = 655 (300 males, 355 females)	≥ 70 years	Community dwellings in one district (Coyoacán)	Modified periodontal screening index measuring probing depth; clinical assessment of periodontal status, including gingivitis, moderate and severe periodontitis, and edentulism	OHIP‐14	The clinical periodontal conditions assessed were not significantly associated with OHRQoL scores in either univariate or multivariate analysis (*p* = 0.305)
Hijryana et al. [50]	Indonesia	Cross‐sectional	*N* = 363 (*n* = 214 ≥ 65 years old)	≥ 65 years (subgroup)	Community health centres in 3 districts of Depok (urban population)	Basic Periodontal Examination (BPE) for initial screening; classification of periodontal status into generalised, localised periodontitis, or healthy. Simplified Oral Hygiene Index (OHI‐S), tooth mobility status, and furcation involvement status	OHIP‐14	Periodontal disease status (presence/absence) was not significantly associated with OHRQoL outcomes (prevalence, severity, or extent of impacts). Tooth mobility was significantly associated with lower OHRQoL across all assessments, including prevalence (odds ratio = 1.87, *p* = 0.009), severity, and extent of impacts
Srisilapanan and Sheiham [51]	Thailand	Cross‐sectional	*N* = 707 (276 males, 431 females)	60–74 years	Community elders in Chiang Mai	Probing depth, attachment loss, number of missing teeth, mobile teeth	OIDP	Tooth loss and periodontal attachment loss was significantly associated with higher OIDP scores (*p* < 0.001). Tooth mobility was also significantly related to greater oral impacts (*p* = 0.05), while probing depth was not (*p* = 0.994)
Hunt et al. [52]	USA	Cross‐sectional	*N* = 440 (208 males, 232 females)	≥ 70 years	Community‐dwelling.	Deepest periodontal pocket, most severe attachment loss, missing teeth	OHIP‐49	Deepest periodontal pocket depth was significantly associated with poorer OHRQoL (*p* < 0.0001). Attachment loss was not a significant predictor after adjustment
Swoboda et al. [53]	USA and Canada	Cross‐sectional	*N* = 733 (326 males, 407 females)	60–75 years	Community‐dwelling older adults	CAL	GOHAI	Sites with CAL ≥ 5 mm were significantly associated with poorer OHRQoL, showing negative correlations with GOHAI scores (*p* < 0.001)
Masood et al. [54]	UK	Cross‐sectional	*N* = 1277 (627 males, 650 females)	≥ 65 years	National older adult population	PPD	OHIP‐14	Indices of periodontal health (having periodontal pocket ≥ 4 mm or periodontal bleeding) showed no statistically significant relationship with OHRQoL
Sheng et al. [55]	China	Cross‐sectional	*N* = 687 (359 males, 328 females)	≥ 60 years	Community‐dwelling older adults	CPI	OHIP‐14	Higher severity of periodontitis correlates with worse OHRQoL but only some QoL outcome measures had statistical significance
Cornejo et al. [56]	Spain	Cross‐sectional	*N* = 194 (56 males, 138 females)	≥ 65 years	Public social health centres for older adults	CPI	GOHAI	Needing periodontal treatment (per CPI) was not significantly associated with poor OHRQoL (PR = 0.92; 95% CI: 0.75–1.13)
Marya et al. [57]	India	Cross‐sectional	*N* = 1200 (746 males, 454 females)	≥ 60 years	Community‐dwelling older adults	Loss of attachment, BOP and mobility	GOHAI	Mobile teeth was the only periodontal variable significantly associated with OHRQoL (*p* < 0.0001). Periodontal pockets, bleeding, and loss of attachment (LOA) showed no statistically significant association with GOHAI scores (all *p* > 0.05)
Shivakumar et al. [58]	India	Cross‐sectional	*N* = 150 (68 males, 82 females)	≥ 65 years	Institutionalised elders in old age homes	CPI	GOHAI	Needing periodontal treatment (as per CPI) was not significantly associated with poor OHRQoL (*p* > 0.05). Participants with mobile teeth had lower mean GOHAI scores than those without mobility (*p* < 0.0001)
Rani et al. [59]	India	Cross‐sectional	*N* = 400 (211 males, 189 females)	≥ 60 years	Institutionalised older adults	CPI and loss of attachment	GOHAI	CPI was only significantly related to OHRQoL in the pain/discomfort domain (*p* = 0.049). Loss of attachment showed highly significant associations across all three GOHAI domains (functional, pain/discomfort, psychosocial), with *p* = 0.000 for each
Göktürk & Uçan Yarkaç, 2018 [60]	Turkey	Cross‐sectional	*N* = 110 (54 males, 56 females)	65–92 years	Older adult patients recruited from the Periodontology Department, Faculty of Dentistry, Gaziosmanpasa University	Plaque Index (PI), Gingival Index (GI), PPD, CAL, BOP, mobility	GOHAI	Greater periodontal disease severity (higher PPD, CAL, BOP, and mobility) was linked to poorer OHRQoL (*p* < 0.005)
Shao et al. [61]	China	Cross‐sectional	*N* = 744 (362 males, 382 females)	65–74 years	Urban and rural communities in Sichuan Province	PPD and loss of attachment	GOHAI	Greater pocket depths is associated with decreased OHRQoL (*p* = 0.440) and greater loss of attachment is associated with decreased OHRQoL (*p* = 0.033)
Sødal et al. [62]	Norway	Cross‐sectional	*N* = 151 (50 males, 101 females)	≥ 65 years	Public dental health clinics in Rogaland County	Periodontal diagnosis per 2017 World Workshop classification (Healthy, Gingivitis, Stage I–IV Periodontitis)	OHIP‐14	Multivariate analysis showed severe periodontitis independently associated with poorer OHRQoL. Significantly higher proportion of those with stage III or IV periodontitis compared to those without periodontitis or stage II periodontitis reported a negative impact on the OHRQoL in the psychological disability domain (*p* < 0.05)
Sukumar et al. [63]	India	Cross‐sectional	*N* = 881 (208 males, 673 females)	≥ 60 years	Irula tribal population, Thiruvallur district, Tamil Nadu	CPITN	GOHAI	Periodontitis had significant associations with impaired OHRQoL (AOR = 1.686, 95% CI: 1.213–2.343, *p* = 0.002)
Zhao et al. [64]	China	Cross‐sectional	*N* = 300 (146 males, 154 females)	60–80 years	Community dwelling	Number of teeth with PPD ≥ 6 mm, and number of teeth with LOA ≥ 6 mm.	Chinese GOHAI	In bivirate analysis number of teeth with PPD ≥ 6 mm was associated with OHRQol whereas PPD was not. In multivariate ANCOVA absence of teeth with LOA ≥ 6 mm (*p* < 0.001), was associated with higher (better) GOHAI
Zhi et al. [65]	China	Cross‐sectional	*N* = 4332 (2174 males, 2158 females)	65–74 years	Community dwelling	CPI (probing pockets and loss of attachment categories)	Mandarin version GOHAI	Presence of periodontal pockets ≥ 6 mm (*B* = −1.050, *p* < 0.001) and attachment loss (*B* = −0.536, *p* < 0.001) were both independently associated with poorer GOHAI scores

Abbreviations: BOP, bleeding on probing; CAL, clinical attachment level; CPI, Community Periodontal Index; CPITN, Community Periodontal Index of Treatment Needs; GOHAI, Geriatric Oral Health Assessment Index; LOA, loss of attachment; OHIP‐14, 14‐item Oral Health Impact Profile; OHIP‐49, 49‐item Oral Health Impact Profile; OHI‐S, Simplified Oral Hygiene Index; OHRQoL, oral health‐related quality of life; OIDP, oral impacts on daily performances; PPD, probing pocket depth; REC, gingival recession.

Periodontal diagnosis was based on PPD in 12 studies, CAL in 8 studies, and CPI/CPITN in 9 studies. Some studies applied combined definitions, for example incorporating both PPD and CAL or using the 2017 AAP/EFP classification. Diagnostic thresholds and disease definitions varied substantially, with PPD cut‐offs ranging from ≥ 4 to ≥ 6 mm, CAL thresholds from ≥ 3 to ≥ 6 mm, and CPI/CPITN codes ranging from 0 to 4. OHRQoL was most frequently measured using the GOHAI (*n* = 14) or OHIP‐14 (*n* = 10), with fewer studies employing the OHIP‐49 (*n* = 3) or OIDP (*n* = 2). Counts are not mutually exclusive.

#### 3.3.2 Main Findings

The association between PPD, CAL, or CPI/CPITN and OHRQoL was inconsistent across studies [41, 50, 54, 56]. Several studies reported significant associations between deeper pockets, greater attachment loss, or higher CPI/CPITN scores and poorer OHRQoL [42, 46, 55, 60, 63, 64, 65, 66]. These associations were observed in both bivariate and multivariable analyses, with some showing domain‐specific impacts, for example: psychological disability on the OHIP‐14 [66], pain/discomfort on the GOHAI [59], and functional limitation on the OHIP‐49 (alongside other domains) [46]; in others, significance was confined to selected OHIP dimensions [55]. Zhao and colleagues [64] reported that attachment loss ≥ 6 mm was significantly associated with lower GOHAI scores, while Zhi et al. [65] found both periodontal pockets ≥ 6 mm and attachment loss independently associated with poorer GOHAI outcomes.

In contrast, an almost equal number of studies reported no statistically significant association between these periodontal measures and OHRQoL [41, 43, 45, 47, 49, 50, 54, 56, 58]. In these, OHRQoL scores did not differ significantly across periodontal status categories after adjusting for potential confounders. Some reported that other clinical variables (e.g., tooth mobility) were more consistently associated with OHRQoL than PPD, CAL, or CPI/CPITN.

A smaller number of studies reported mixed or partial associations. For example, León and colleagues [46] found higher OHIP‐49 scores among those requiring complex periodontal treatment compared to those with simpler needs, while Sheng and co‐workers [55] reported significant associations for only some OHRQoL dimensions. Rani et al. [59] found loss of attachment significantly associated with all GOHAI domains, while CPI was only significant in the pain/discomfort domain.

#### 3.3.3 Summary OHRQoL Outcome

Across these 25 studies, the evidence linking PPD, CAL, or CPI/CPITN to poorer OHRQoL in older adults was mixed. While several studies demonstrated statistically significant associations, an almost equal proportion reported no relationship, even in large community‐based samples. Heterogeneity in diagnostic criteria, disease severity thresholds, OHRQoL instruments, and analytic approaches likely contributed to these divergent findings. Overall, while deeper pockets, greater attachment loss, and higher CPI/CPITN scores are sometimes associated with poorer OHRQoL, this relationship is not consistent across populations or study designs.

### 3.4 Risk of Bias

Risk of bias analysis is presented in Data S3. Across the 18 longitudinal studies assessing clinical outcomes, study quality was variable, with most rated as fair and only a minority meeting criteria for good quality. A common limitation was the inconsistent definition of exposure variables, with notable variation in diagnostic criteria and classification thresholds across studies. Although validated clinical measures were typically used, control for confounding factors varied widely, and loss to follow‐up was often poorly documented. These issues highlight the need for cautious interpretation of the findings.

In the 25 cross‐sectional studies investigating OHRQoL, methodological quality showed a similar pattern, with the majority rated as fair and few achieving a good rating. Selection bias was frequently introduced by non‐probability sampling methods and limited information on non‐respondents. Most studies used standardised clinical and OHRQoL instruments; however, confounder adjustment was inconsistent and missing data were rarely addressed. These methodological weaknesses also limit the certainty of the conclusions drawn.

## 4 Discussion

This systematic review examined the impact of periodontitis on clinical oral health outcomes and OHRQoL in adults aged ≥ 60 years, drawing on evidence from 43 studies across diverse geographic settings. For clinical outcomes, the evidence was strikingly consistent across 18 longitudinal studies. Deeper baseline periodontal pockets, greater CAL, and higher CPI/CPITN scores consistently predicted adverse outcomes over follow‐up periods ranging from 18 months to 12 years [24, 31, 34, 37]. These included higher risks of tooth loss, often with odds ratios exceeding 2.0 for severe baseline attachment loss [31, 34], further periodontal breakdown [26, 30, 36], and deterioration in measures such as swallowing function and oral frailty [32, 35]. However, outcomes such as swallowing impairment and oral frailty, while highly relevant in gerodontology, were examined in only a small number of studies, and the evidence base for these outcomes therefore remains limited. Associations persisted across populations, diagnostic criteria, and analytical approaches, demonstrating a robust dose–response relationship between baseline periodontitis severity and subsequent deterioration [31, 33, 37]. These findings are consistent with previous systematic reviews in broader adult populations, which have confirmed periodontitis as a major predictor of tooth loss and further attachment loss [67, 68]. Our age‐specific synthesis extends these conclusions to adults aged 60 years and older, emphasising that severe periodontitis in later life carries a high risk of both tooth loss and continued periodontal tissue destruction.

In contrast, the evidence linking periodontitis to oral health‐related quality of life was inconsistent across the 25 included cross‐sectional studies. While several studies demonstrated statistically significant associations between measures of periodontitis and poorer OHRQoL [42, 46, 48, 63, 64, 65], an almost equal proportion reported no association, even in large community‐based samples [49, 54, 56]. These variable findings persisted despite the use of validated instruments such as OHIP and GOHAI, and were observed across diverse geographic and demographic settings [45, 60, 62]. The inconsistency aligns with previous systematic reviews in mixed‐age populations, which have reported modest pooled associations between periodontitis and OHRQoL but with substantial heterogeneity and evidence of effect modification by factors such as comorbidities and tooth loss [18, 69]. Our review indicates that even within older adult populations, periodontitis is not consistently associated with OHRQoL, suggesting that downstream consequences such as tooth loss and functional impairment may have a more direct influence in shaping quality of life in later life [51, 57].

A major challenge in synthesising these findings was the substantial heterogeneity in periodontitis case definitions and diagnostic approaches. Studies used varying combinations of PPD, CAL, and CPI/CPITN measures, with different thresholds for defining disease presence and severity. CAL, although a strong predictor of future tooth loss and periodontal breakdown, cannot distinguish active from stabilised disease or from inflammation‐free recession, limiting its value in assessing current health status and immediate treatment needs [25, 29]. This variability in disease definition was compounded by differences in outcome measurement, with clinical studies using varied definitions of tooth loss (e.g., ≥ 1, ≥ 3, ≥ 4 teeth) [27, 33, 39], different criteria for periodontal disease progression [26, 36], and diverse measures for functional decline [32, 35]. OHRQoL studies likewise employed a range of instruments with differing scoring approaches (OHIP‐14, OHIP‐49, GOHAI, OIDP) [41, 43, 47]. Follow‐up durations ranged from 18 months to 12 years, and populations included both community‐dwelling and institutionalised older adults [38, 58]. This multidimensional heterogeneity meant that meta‐analysis was not methodologically appropriate, as pooled estimates would not have been clinically meaningful or statistically valid.

The inconsistency in observed associations between periodontitis and OHRQoL likely reflects the complexity of pathways through which periodontitis impacts patient‐reported outcomes. In the included longitudinal studies, periodontitis consistently preceded tooth loss, periodontal breakdown, and functional decline [24, 31, 35], yet few studies directly examined whether these sequelae mediated OHRQoL impacts. Where quality of life measures were available, tooth loss, mobility, and functional impairment tended to show stronger and more consistent associations with OHRQoL than periodontal parameters themselves [45, 59]. However, recent cross‐sectional evidence [65] indicates that when disease reaches more severe thresholds, periodontal pockets and attachment loss can also be independently associated with poorer OHRQoL. Taken together, these findings suggest that the impact of periodontitis on OHRQoL is usually indirect, operating through consequences such as tooth loss and functional decline, though a direct effect may also arise in advanced disease.

The strengths of this review include a comprehensive search strategy, rigorous study selection and data extraction, and a focus on adults aged ≥ 60 years, the group most affected by severe periodontitis and its consequences. Including both clinical and patient‐reported outcomes provides a more complete understanding of the impact of periodontitis in later life. However, certain limitations should be acknowledged. Other oral conditions such as coronal and root caries were not included as outcomes, although they are important competing causes of tooth loss in older adults [70, 71]. Specific manifestations of periodontitis such as tooth mobility and furcation involvement were also not systematically captured, despite their potential relevance to patient experiences [48]. For OHRQoL, the fundamental difficulty lies in disentangling the effects of periodontitis from those of tooth loss, given the strong causal link between the two [31, 39]. Differences in sampling, with most clinical outcome studies involving community‐dwelling participants and some OHRQoL studies including institutionalised populations, may also have influenced the patterns observed [38, 58]. Risk of bias assessments indicated that most included studies were of fair methodological quality, with frequent limitations in sampling strategies, exposure definitions, confounder adjustment, and follow‐up completeness. These methodological concerns further reinforce the need for cautious interpretation of the observed associations. Finally, meta‐analysis was not feasible due to substantial heterogeneity in periodontal case definitions, outcome measures, follow‐up periods and analytical approaches, which limited the ability to generate pooled quantitative estimates.

The consistent evidence linking periodontitis severity to adverse clinical outcomes supports the prioritisation of periodontal assessment and management in older adults. Dose–response relationships indicate that even moderate periodontitis confers substantial risk for future tooth loss and functional decline [33, 37], underscoring the importance of early detection and ongoing maintenance. In discussing treatment with older patients, clinicians should emphasise the prevention of downstream consequences, particularly tooth loss and functional impairment, rather than periodontal parameters alone [28, 32]. Future research should focus on longitudinal designs that can map the sequence from periodontitis progression to tooth loss and quality of life changes, incorporating measures of current disease activity and functional status. Trials evaluating the effectiveness of periodontitis interventions in preventing tooth loss and preserving function and OHRQoL in older adults would provide essential evidence for guiding clinical decision‐making.

## 5 Conclusions

This systematic review of 43 studies revealed consistent evidence that periodontitis predicts adverse clinical oral health outcomes in older adults, but inconsistent associations with oral health‐related quality of life. Across 18 longitudinal studies, deeper baseline periodontal pockets, greater clinical attachment loss, and higher CPI/CPITN scores consistently predicted tooth loss, further periodontal breakdown, and functional decline over follow‐up periods ranging from 18 months to 12 years. In contrast, 25 cross‐sectional studies examining the relationship between periodontitis and OHRQoL showed mixed findings, with roughly equal numbers reporting significant associations and no relationship. These findings suggest that while periodontitis is a robust predictor of clinical deterioration in older adults, its impact on quality of life may be mediated primarily through downstream consequences such as tooth loss and functional impairment rather than periodontal status alone.

It is important to distinguish between the nature of the evidence underpinning these findings. The associations between periodontitis and adverse clinical oral health outcomes are supported predominantly by longitudinal studies, allowing prediction of tooth loss, periodontal breakdown, and functional decline over time. In contrast, evidence linking periodontitis to oral health‐related quality of life is derived largely from cross‐sectional studies and should therefore be interpreted as associative rather than causal, with potential bidirectionality and residual confounding. Further longitudinal research is needed to clarify the temporal relationships between periodontal disease progression, tooth loss, and quality of life changes in older populations.

## Author Contributions

**Lewis Winning:** conceptualisation, methodology, project administration, data curation, writing – original draft. **Lewis Winning**, **Kristina Bertl**, **Ioannis Polyzois**, **Peter Harrison**, **Yvonne Van der Waal**, **Rawan Kahatab:** screening, data extraction, interpretation of findings, writing – review and editing. **Lewis Winning**, **Rawan Kahatab:** risk of bias assessment. **Gerry McKenna:** supervision, interpretation of findings, writing – review and editing.

## Funding

The authors have nothing to report.

## Disclosure

PROSPERO Registration: https://www.crd.york.ac.uk/PROSPERO/view/CRD420251010568.

## Conflicts of Interest

The authors declare no conflicts of interest.

## Supporting information


**Data S1:** Provides the full electronic search strategies used for all databases, including PubMed, MEDLINE, Embase, Scopus, and the Cochrane Library.

**Data S2:** Presents a table of studies excluded following full‐text review, with reasons for exclusion.

**Data S3:** Reports the risk of bias assessments for included cohort and cross‐sectional studies using the Newcastle–Ottawa Scale and a modified Newcastle–Ottawa Scale, respectively.

## Data Availability

The data that support the findings of this study are available on request from the corresponding author.

## References

[1] M. A. Peres , L. M. D. Macpherson , R. J. Weyant , et al., “Oral Diseases: A Global Public Health Challenge,” Lancet 394, no. 10194 (2019): 249–260. 31327369 10.1016/S0140-6736(19)31146-8

[2] E. Bernabe , W. Marcenes , C. R. Hernandez , et al., “Global, Regional, and National Levels and Trends in Burden of Oral Conditions From 1990 to 2017: A Systematic Analysis for the Global Burden of Disease 2017 Study,” Journal of Dental Research 99, no. 4 (2020): 362–373. 32122215 10.1177/0022034520908533 PMC7088322

[3] L. Wu , C. M. Huang , Q. Wang , J. Wei , L. Xie , and C. Y. Hu , “Burden of Severe Periodontitis: New Insights Based a Systematic Analysis From the Global Burden of Disease Study 2021,” BMC Oral Health 25, no. 1 (2025): 861. 40450275 10.1186/s12903-025-06271-0 PMC12125741

[4] P. I. Eke , L. Wei , W. S. Borgnakke , et al., “Periodontitis Prevalence in Adults ≥ 65 Years of Age, in the USA,” Periodontology 2000 72, no. 1 (2016): 76–95. 27501492 10.1111/prd.12145 PMC8223257

[5] G. G. Nascimento , S. Alves‐Costa , and M. Romandini , “Burden of Severe Periodontitis and Edentulism in 2021, With Projections up to 2050: The Global Burden of Disease 2021 Study,” Journal of Periodontal Research 59, no. 5 (2024): 823–867. 39192495 10.1111/jre.13337

[6] L. Chambrone , D. Chambrone , L. A. Lima , and L. A. Chambrone , “Predictors of Tooth Loss During Long‐Term Periodontal Maintenance: A Systematic Review of Observational Studies,” Journal of Clinical Periodontology 37, no. 7 (2010): 675–684. 20528960 10.1111/j.1600-051X.2010.01587.x

[7] A. E. Gerritsen , P. F. Allen , D. J. Witter , E. M. Bronkhorst , and N. H. Creugers , “Tooth Loss and Oral Health‐Related Quality of Life: A Systematic Review and Meta‐Analysis,” Health and Quality of Life Outcomes 8 (2010): 126. 21050499 10.1186/1477-7525-8-126 PMC2992503

[8] F. Müller , Y. Shimazaki , F. Kahabuka , and M. Schimmel , “Oral Health for an Ageing Population: The Importance of a Natural Dentition in Older Adults,” International Dental Journal 67, no. 2 (2017): 7–13. 29023743 10.1111/idj.12329 PMC9379181

[9] T. Lahoud , A. Y. Yu , and S. King , “Masticatory Dysfunction in Older Adults: A Scoping Review,” Journal of Oral Rehabilitation 50, no. 8 (2023): 724–737. 37183339 10.1111/joor.13493

[10] L. Winning , C. C. Patterson , C. E. Neville , F. Kee , and G. J. Linden , “Periodontitis and Incident Type 2 Diabetes: A Prospective Cohort Study,” Journal of Clinical Periodontology 44, no. 3 (2017): 266–274. 28036104 10.1111/jcpe.12691

[11] L. Winning , C. C. Patterson , K. M. Cullen , F. Kee , and G. J. Linden , “Chronic Periodontitis and Reduced Respiratory Function,” Journal of Clinical Periodontology 46, no. 3 (2019): 266–275. 30712268 10.1111/jcpe.13076

[12] L. Winning , C. C. Patterson , K. Linden , et al., “Periodontitis and Risk of Prevalent and Incident Coronary Heart Disease Events,” Journal of Clinical Periodontology 47, no. 12 (2020): 1446–1456. 32998173 10.1111/jcpe.13377

[13] L. Winning , C. C. Patterson , K. Linden , K. M. Cullen , F. Kee , and G. J. Linden , “Systemic Inflammation and the Relationship Between Periodontitis, Edentulism, and All‐Cause Mortality: A 17‐Year Prospective Cohort Study,” Journal of Clinical Periodontology 48, no. 9 (2021): 1260–1269. 34109647 10.1111/jcpe.13510

[14] M. S. Tonetti , H. Greenwell , and K. S. Kornman , “Staging and Grading of Periodontitis: Framework and Proposal of a New Classification and Case Definition,” Journal of Clinical Periodontology 45, no. 20 (2018): S149–s161. 29926495 10.1111/jcpe.12945

[15] A. Savage , K. A. Eaton , D. R. Moles , and I. Needleman , “A Systematic Review of Definitions of Periodontitis and Methods That Have Been Used to Identify This Disease,” Journal of Clinical Periodontology 36, no. 6 (2009): 458–467. 19508246 10.1111/j.1600-051X.2009.01408.x

[16] Z. S. Natto , R. H. Abu Ahmad , L. T. Alsharif , et al., “Chronic Periodontitis Case Definitions and Confounders in Periodontal Research: A Systematic Assessment,” BioMed Research International 2018 (2018): 4578782. 30622957 10.1155/2018/4578782 PMC6304204

[17] L. Ke , G. Nogueira , and W. M. Thomson , “Influence of Case Definitions on Epidemiological Estimates of Periodontitis Prevalence and Its Associations With Smoking and OHRQoL,” Community Dentistry and Oral Epidemiology 51, no. 2 (2023): 194–200. 35076110 10.1111/cdoe.12726

[18] C. C. D. Agnese , C. Schöffer , K. Z. Kantorski , F. B. Zanatta , C. Susin , and R. P. Antoniazzi , “Periodontitis and Oral Health‐Related Quality of Life: A Systematic Review and Meta‐Analysis,” Journal of Clinical Periodontology 52, no. 3 (2025): 408–420. 39343995 10.1111/jcpe.14074

[19] D. Moher , A. Liberati , J. Tetzlaff , and D. G. Altman , “Preferred Reporting Items for Systematic Reviews and Meta‐Analyses: The PRISMA Statement,” PLoS Medicine 6, no. 7 (2009): e1000097. 19621072 10.1371/journal.pmed.1000097 PMC2707599

[20] M. J. Page , J. E. McKenzie , P. M. Bossuyt , et al., “The PRISMA 2020 Statement: An Updated Guideline for Reporting Systematic Reviews,” BMJ 372 (2021): n71. 33782057 10.1136/bmj.n71 PMC8005924

[21] G. A. Wells , B. Shea , D. O'Connell , et al., The Newcastle–Ottawa Scale (NOS) for Assessing the Quality of Non‐Randomised Studies in Meta‐Analyses (Ottawa Hospital Research Institute, 2011).

[22] J. R. Landis and G. G. Koch , “The Measurement of Observer Agreement for Categorical Data,” Biometrics 33, no. 1 (1977): 159–174. 843571

[23] J. D. Beck , G. G. Koch , and S. Offenbacher , “Attachment Loss Trends Over 3 Years in Community‐Dwelling Older Adults,” Journal of Periodontology 65, no. 8 (1994): 737–743. 7965548 10.1902/jop.1994.65.8.737

[24] J. D. Beck , T. Sharp , G. G. Koch , and S. Offenbacher , “A 5‐Year Study of Attachment Loss and Tooth Loss in Community‐Dwelling Older Adults,” Journal of Periodontal Research 32, no. 6 (1997): 516–523. 9379319 10.1111/j.1600-0765.1997.tb00567.x

[25] J. D. Beck , T. Sharp , G. G. Koch , and S. Offenbacher , “A Study of Attachment Loss Patterns in Survivor Teeth at 18 Months, 36 Months and 5 Years in Community‐Dwelling Older Adults,” Journal of Periodontal Research 32, no. 6 (1997): 497–505. 9379317 10.1111/j.1600-0765.1997.tb00565.x

[26] L. F. Brown , J. D. Beck , and R. G. Rozier , “Incidence of Attachment Loss in Community‐Dwelling Older Adults,” Journal of Periodontology 65, no. 4 (1994): 316–323. 8195975 10.1902/jop.1994.65.4.316

[27] T. Gonda , M. I. MacEntee , H. A. Kiyak , G. R. Persson , R. E. Persson , and C. Wyatt , “Predictors of Multiple Tooth Loss Among Socioculturally Diverse Elderly Subjects,” International Journal of Prosthodontics 26, no. 2 (2013): 127–134. 23476905 10.11607/ijp.2893

[28] A. J. Hassel , V. Safaltin , S. Grill , et al., “Risk Factors for Tooth Loss in Middle and Older Age After up to 10 Years: An Observational Cohort Study,” Archives of Oral Biology 86 (2018): 7–12. 29132069 10.1016/j.archoralbio.2017.11.001

[29] T. Hirotomi , A. Yoshihara , M. Yano , Y. Ando , and H. Miyazaki , “Longitudinal Study on Periodontal Conditions in Healthy Elderly People in Japan,” Community Dentistry and Oral Epidemiology 30, no. 6 (2002): 409–417. 12453111 10.1034/j.1600-0528.2002.00005.x

[30] T. Hirotomi , A. Yoshihara , H. Ogawa , and H. Miyazaki , “Tooth‐Related Risk Factors for Periodontal Disease in Community‐Dwelling Elderly People,” Journal of Clinical Periodontology 37, no. 6 (2010): 494–500. 20507372 10.1111/j.1600-051X.2010.01565.x

[31] T. Hirotomi , A. Yoshihara , H. Ogawa , and H. Miyazaki , “Tooth‐Related Risk Factors for Tooth Loss in Community‐Dwelling Elderly People,” Community Dentistry and Oral Epidemiology 40, no. 2 (2012): 154–163. 22044265 10.1111/j.1600-0528.2011.00648.x

[32] K. Iwai , T. Azuma , T. Yonenaga , et al., “Predictive Factors Associated With Future Decline in Swallowing Function Among Japanese Older People Aged ≥ 75 Years,” International Journal of Environmental Research and Public Health 21, no. 6 (2024): 674. 38928921 10.3390/ijerph21060674 PMC11203831

[33] Y. Mihara , K. I. Matsuda , T. Takahashi , et al., “Occlusal Support Predicts Tooth Loss in Older Japanese People,” Community Dentistry and Oral Epidemiology 48, no. 2 (2020): 163–170. 31876315 10.1111/cdoe.12515

[34] H. Nilsson , J. Sanmartin Berglund , and S. Renvert , “Longitudinal Evaluation of Periodontitis and Tooth Loss Among Older Adults,” Journal of Clinical Periodontology 46, no. 10 (2019): 1041–1049. 31294471 10.1111/jcpe.13167

[35] M. Nishimoto , T. Tanaka , H. Hirano , et al., “Severe Periodontitis Increases the Risk of Oral Frailty: A Six‐Year Follow‐Up Study From Kashiwa Cohort Study,” Geriatrics 8, no. 1 (2023): 25. 36826367 10.3390/geriatrics8010025 PMC9956982

[36] H. Ogawa , A. Yoshihara , T. Hirotomi , Y. Ando , and H. Miyazaki , “Risk Factors for Periodontal Disease Progression Among Elderly People,” Journal of Clinical Periodontology 29, no. 7 (2002): 592–597. 12354083 10.1034/j.1600-051x.2002.290702.x

[37] H. Sato , K. Hatta , Y. Murotani , et al., “Predictive Factors for Tooth Loss in Older Adults Vary According to Occlusal Support: A 6‐Year Longitudinal Survey From the SONIC Study,” Journal of Dentistry 121 (2022): 104088. 35259445 10.1016/j.jdent.2022.104088

[38] Y. Shimazaki , I. Soh , T. Koga , H. Miyazaki , and T. Takehara , “Risk Factors for Tooth Loss in the Institutionalised Elderly: A Six‐Year Cohort Study,” Community Dental Health 20, no. 2 (2003): 123–127. 12828274

[39] G. D. Slade , S. A. Gansky , and A. J. Spencer , “Two‐Year Incidence of Tooth Loss Among South Australians Aged 60+ Years,” Community Dentistry and Oral Epidemiology 25, no. 6 (1997): 429–437. 9429816 10.1111/j.1600-0528.1997.tb01734.x

[40] J. J. Warren , C. A. Watkins , H. J. Cowen , J. S. Hand , S. M. Levy , and R. A. Kuthy , “Tooth Loss in the Very Old: 13–15‐Year Incidence Among Elderly Iowans,” Community Dentistry and Oral Epidemiology 30, no. 1 (2002): 29–37. 11918573 10.1034/j.1600-0528.2002.300105.x

[41] D. Agustina , L. Hanindriyo , B. E. Chrismawaty , and F. Naritasari , “Oral Conditions as Risk Factors for Low Oral Health‐Related Quality of Life Among the Elderly Population in Yogyakarta, Indonesia,” European Journal of Dentistry 17, no. 2 (2023): 504–510. 36513333 10.1055/s-0042-1757566 PMC10329552

[42] J. Botelho , V. Machado , L. Proença , et al., “Perceived Xerostomia, Stress and Periodontal Status Impact on Elderly Oral Health‐Related Quality of Life: Findings From a Cross‐Sectional Survey,” BMC Oral Health 20, no. 1 (2020): 199. 32650751 10.1186/s12903-020-01183-7 PMC7350690

[43] J. O. Guevara‐Canales , R. Morales‐Vadillo , S. J. Sacsaquispe‐Contreras , D. E. Alberca‐Ramos , H. Morgenstern‐Orezzoli , and C. E. Cava‐Vergiú , “Association Between Self‐Perceived Oral Health and Clinical Indicators,” Oral Health & Preventive Dentistry 16, no. 1 (2018): 33–41. 29335684 10.3290/j.ohpd.a39685

[44] O. Gokturk and F. U. Yarkac , “Comparison of Two Measures to Determine the Oral Health‐Related Quality of Life in Elders With Periodontal Disease,” Community Dental Health 36, no. 2 (2019): 143–149. 31070876 10.1922/CDH_4387Gokturk07

[45] C. M. Marya , H. S. Grover , S. Tandon , P. Taneja , A. Gupta , and V. Marya , “Gender‐Wise Comparison of Oral Health‐Related Quality of Life and Its Relationship With Periodontal Status Among the Indian Elderly,” Journal of Indian Society of Periodontology 24, no. 1 (2020): 72–79. 31983849 10.4103/jisp.jisp_156_19 PMC6961442

[46] S. León , D. Bravo‐Cavicchioli , R. A. Giacaman , G. Correa‐Beltrán , and C. Albala , “Validation of the Spanish Version of the Oral Health Impact Profile to Assess an Association Between Quality of Life and Oral Health of Elderly Chileans,” Gerodontology 33, no. 1 (2016): 97–105. 24612284 10.1111/ger.12124

[47] T. Kato , I. Abrahamsson , U. Wide , and M. Hakeberg , “Periodontal Disease Among Older People and Its Impact on Oral Health‐Related Quality of Life,” Gerodontology 35, no. 4 (2018): 382–390. 30043453 10.1111/ger.12363

[48] A. Rekhi , C. M. Marya , S. S. Oberoi , R. Nagpal , C. Dhingra , and S. Kataria , “Periodontal Status and Oral Health‐Related Quality of Life in Elderly Residents of Aged Care Homes in Delhi,” Geriatrics & Gerontology International 16, no. 4 (2016): 474–480. 25952758 10.1111/ggi.12494

[49] R. C. Castrejón‐Pérez , S. A. Borges‐Yáñez , M. E. Irigoyen‐Camacho , and L. P. Cruz‐Hervert , “Negative Impact of Oral Health Conditions on Oral Health Related Quality of Life of Community Dwelling Elders in Mexico City, a Population Based Study,” Geriatrics & Gerontology International 17, no. 5 (2017): 744–752. 27150729 10.1111/ggi.12780

[50] M. Hijryana , M. MacDougall , N. Ariani , P. Saksono , L. S. Kusdhany , and A. W. G. Walls , “Periodontal Disease and Oral Health‐Related Quality of Life in the Older Population in Indonesia,” JDR Clinical & Translational Research 7, no. 3 (2022): 277–288. 34282670 10.1177/23800844211021391 PMC9203662

[51] P. Srisilapanan and A. Sheiham , “The Prevalence of Dental Impacts on Daily Performances in Older People in Northern Thailand,” Gerodontology 18, no. 2 (2001): 102–108. 11794735 10.1111/j.1741-2358.2001.00102.x

[52] R. J. Hunt , G. D. Slade , and R. P. Strauss , “Differences Between Racial Groups in the Impact of Oral Disorders Among Older Adults in North Carolina,” Journal of Public Health Dentistry 55, no. 4 (1995): 205–209. 8551459 10.1111/j.1752-7325.1995.tb02371.x

[53] J. Swoboda , H. A. Kiyak , R. E. Persson , et al., “Predictors of Oral Health Quality of Life in Older Adults,” Special Care in Dentistry 26, no. 4 (2006): 137–144. 16927735 10.1111/j.1754-4505.2006.tb01714.x

[54] M. Masood , T. Newton , N. N. Bakri , T. Khalid , and Y. Masood , “The Relationship Between Oral Health and Oral Health Related Quality of Life Among Elderly People in United Kingdom,” Journal of Dentistry 56 (2017): 78–83. 27825838 10.1016/j.jdent.2016.11.002

[55] X. Sheng , X. Xiao , X. Song , L. Qiao , X. Zhang , and H. Zhong , “Correlation Between Oral Health and Quality of Life Among the Elderly in Southwest China From 2013 to 2015,” Medicine 97, no. 21 (2018): e10777. 29794757 10.1097/MD.0000000000010777 PMC6392902

[56] M. Cornejo , G. Pérez , K. C. de Lima , E. Casals‐Peidro , and C. Borrell , “Oral Health‐Related Quality of Life in Institutionalized Elderly in Barcelona (Spain),” Medicina Oral, Patología Oral y Cirugía Bucal 18, no. 2 (2013): e285–e292. 23385501 10.4317/medoral.18280 PMC3613882

[57] C. M. Marya , H. S. Grover , S. Tandon , A. Gupta , R. Nagpal , and P. Taneja , “Oral Health Indicators of Oral Health Related Quality of Life Among Indian Elderly: A Cross‐Sectional Study,” Indian Journal of Dental Research 32, no. 3 (2021): 316–322. 35229770 10.4103/ijdr.IJDR_81_19

[58] K. M. Shivakumar , S. Patil , V. Kadashetti , and V. Raje , “Oral Health‐Related Quality of Life of Institutionalized Elderly in Satara District, India,” Journal of Datta Meghe Institute of Medical Sciences University 13, no. 4 (2018): 183–189.

[59] V. Rani , N. Benjamin , A. Abhilash , M. Bhasin , R. Bhamare , and S. Sinha , “Oral Health Status, Dietary Intake, and Oral Health‐Related Quality of Life Among Institutionalized Elderly in Bangalore,” Journal of Pharmacy & Bioallied Sciences 16, no. 3 (2024): S2347–S2349. 39346388 10.4103/jpbs.jpbs_234_24 PMC11426689

[60] Ö. Göktürk and F. Uçan Yarkaç , “Assessment of Oral Health‐Related Quality of Life Among Elderly Patients With Periodontal Disease,” Türkish Geriatric Dergisi 21, no. 3 (2018): 313–323.

[61] R. Shao , T. Hu , Y. S. Zhong , et al., “Socio‐Demographic Factors, Dental Status and Health‐Related Behaviors Associated With Geriatric Oral Health‐Related Quality of Life in Southwestern China,” Health and Quality of Life Outcomes 16, no. 1 (2018): 98. 29784008 10.1186/s12955-018-0925-8 PMC5963059

[62] A. T. T. Sødal , R. Skudutyte‐Rysstad , M. T. Diep , O. C. Koldsland , and L. H. Hove , “Periodontitis in a 65‐Year‐Old Population: Risk Indicators and Impact on Oral Health‐Related Quality of Life,” BMC Oral Health 22, no. 1 (2022): 640. 36566179 10.1186/s12903-022-02662-9 PMC9789555

[63] M. B. A. Sukumar , R. M. Peter , and A. Joseph , “Tooth Morbidity and Its Impact on Oral Related Quality of Life in Elderly Tribal Population‐The Irula Experience,” BMC Oral Health 25, no. 1 (2025): 252. 39966845 10.1186/s12903-025-05628-9 PMC11834633

[64] L. Zhao , H. C. Lin , E. C. Lo , and M. C. Wong , “Clinical and Socio‐Demographic Factors Influencing the Oral Health‐Related Quality of Life of Chinese Elders,” Community Dental Health 28, no. 3 (2011): 206–210. 21916355

[65] Q. H. Zhi , Y. Si , X. Wang , et al., “Determining the Factors Associated With Oral Health‐Related Quality of Life in Chinese Elders: Findings From the Fourth National Survey,” Community Dentistry and Oral Epidemiology 50, no. 4 (2022): 311–320. 34213027 10.1111/cdoe.12674

[66] A. T. T. Sødal , L. H. Hove , R. Skudutyte‐Rysstad , and O. C. Koldsland , “Periodontitis and Oral Health‐Related QoL in a 65‐Year‐Old Population,” Journal of Clinical Periodontology 49 (2022): 128.

[67] I. Needleman , R. Garcia , N. Gkranias , et al., “Mean Annual Attachment, Bone Level, and Tooth Loss: A Systematic Review,” Journal of Clinical Periodontology 45, no. 20 (2018): S112–S129. 29926483 10.1111/jcpe.12943

[68] O. Helal , G. Göstemeyer , J. Krois , K. Fawzy El Sayed , C. Graetz , and F. Schwendicke , “Predictors for Tooth Loss in Periodontitis Patients: Systematic Review and Meta‐Analysis,” Journal of Clinical Periodontology 46, no. 7 (2019): 699–712. 31025366 10.1111/jcpe.13118

[69] S. L. Buset , C. Walter , A. Friedmann , R. Weiger , W. S. Borgnakke , and N. U. Zitzmann , “Are Periodontal Diseases Really Silent? A Systematic Review of Their Effect on Quality of Life,” Journal of Clinical Periodontology 43, no. 4 (2016): 333–344. 26810308 10.1111/jcpe.12517

[70] J. E. Frencken , P. Sharma , L. Stenhouse , D. Green , D. Laverty , and T. Dietrich , “Global Epidemiology of Dental Caries and Severe Periodontitis—A Comprehensive Review,” Journal of Clinical Periodontology 44, no. 18 (2017): S94–S105. 28266116 10.1111/jcpe.12677

[71] P. Romandini , C. Marruganti , W. G. Romandini , M. Sanz , S. Grandini , and M. Romandini , “Are Periodontitis and Dental Caries Associated? A Systematic Review With Meta‐Analyses,” Journal of Clinical Periodontology 51, no. 2 (2024): 145–157. 38084804 10.1111/jcpe.13910

