# Supplementary material for: The Impact of Periodontitis on Oral Health Outcomes in Older Adults: A Systematic Review
Source: Gerodontology. 2026 Jan 16;43(3):318–38. doi: 10.1111/ger.70048 (PMC13498920; doi:10.1111/ger.70048)
Supplement: Supplementary file 1 [file GER-43-318-s001.docx]

**Supplementary Information S1**

**Full Seach terms for each database (May 2025)**

**PubMed**

1. “Dental Care for Aged”[MeSH Terms]
2. ("Elder*"[Title/Abstract] OR "Geriatric Population*"[Title/Abstract] OR "Geriatric Patient*"[Title/Abstract] OR “Aging Population"[Title/Abstract] OR “Aged Population"[Title/Abstract] OR "Aged 60"[Title/Abstract] OR "60 year*"[Title/Abstract] OR "Aged 65"[Title/Abstract] OR "65 year*"[Title/Abstract] OR "Gerodont*"[Title/Abstract] OR "Aged 70"[Title/Abstract] OR "70 year*"[Title/Abstract] OR "Aged 75"[Title/Abstract] OR "75 year*"[Title/Abstract] OR "Aged 80"[Title/Abstract] OR "80 year*"[Title/Abstract] OR “Senior*"[Title/Abstract] OR “Care-depend*"[Title/Abstract] OR "Older Adults"[Title/Abstract] OR “Older People” [Title/Abstract])
3. #1 OR #2
4. “Periodontitis”[MeSH Terms]
5. "periodont*"[Title/Abstract]
6. “Periodontal Index”[MeSH Terms]
7. #4 OR #5 OR #6
8. "Oral Health"[MeSH Terms]
9. "Disease Progression"[MeSH Terms]
10. “Tooth Loss”[MeSH Terms]
11. “Jaw, Edentulous”[MeSH Terms]
12. “Tooth Extraction”[MeSH Terms]
13. "Periodontal Atrophy"[Mesh]
14. ("disease progression"[Title/Abstract] OR "attachment loss"[Title/Abstract] OR "bone loss"[Title/Abstract] OR "periodontal progression"[Title/Abstract] OR “Plaque Index"[Title/Abstract] OR “CPITN"[Title/Abstract] OR "Surviv*"[Title/Abstract] OR "tooth loss"[Title/Abstract] OR "edent*"[Title/Abstract] OR "missing teeth"[Title/Abstract])
15. “Tooth extraction”[TIAB:~2] OR “Tooth removal”[TIAB:~2]
16. "Mastication"[MeSH Terms]
17. ("chew*"[Title/Abstract] OR "masticat*"[Title/Abstract] OR "speech"[Title/Abstract] OR "swallow*"[Title/Abstract] OR "function*"[Title/Abstract] OR "eating"[Title/Abstract] OR "comfort"[Title/Abstract])
18. "Quality of Life"[MeSH Terms]
19. ("oral health-related quality of life"[Title/Abstract] OR "OHRQoL"[Title/Abstract] OR "Oral Health Impact Profile"[Title/Abstract] OR "OHIP"[Title/Abstract] OR "Oral Impact on Daily Performances"[Title/Abstract] OR "OIDP"[Title/Abstract])
20. #8 OR #9 OR #10 OR #11 OR #12 OR #13 OR #14 OR #15 OR #16 OR #17 OR #18 OR #19
21. #3 AND #7 AND #20

**Results: 2,102**

**Medline (Ovid)**

1. exp "Dental Care for Aged"/
2. ("Elder*" OR "Geriatric Population*" OR "Geriatric Patient*" OR “Aging Population" OR “Aged Population" OR "Aged 60" OR "60 year*" OR "Aged 65" OR "65 year*" OR "Gerodont*" OR "Aged 70" OR "70 year*" OR "Aged 75" OR "75 year*" OR "Aged 80" OR "80 year*" OR “Senior*" OR “Care-depend*" OR "Older Adults" OR “Older People”).ti,ab
3. 1-2/OR
4. exp Periodontitis/
5. "periodont*”.ti,ab
6. exp Periodontal Index/
7. 4-6/OR
8. exp Oral Health/
9. exp Disease Progression/
10. exp Tooth Loss/
11. exp Jaw, Edentulous/
12. Exp Tooth extraction/
13. exp Periodontal Atrophy/
14. ("disease progression" OR "attachment loss" OR "bone loss" OR "periodontal progression" OR “Plaque Index" OR “CPITN" OR "Surviv*" OR "tooth loss" OR "edent*" OR "missing teeth").ti,ab
15. (tooth adj2 (extract* or remov*)).ti,ab.
16. exp Mastication/
17. ("chew*" OR "masticat*" OR "speech" OR "swallow*" OR "function*" OR "eating" OR "comfort").ti,ab
18. exp "Quality of Life"/
19. ("oral health-related quality of life" OR "OHRQoL" OR "Oral Health Impact Profile" OR "OHIP" OR "Oral Impact on Daily Performances" OR "OIDP").ti,ab
20. 8-19/OR
21. 3 AND 7 AND 20

**Results: 2,050**

**Embase**

1. ("Elder*" OR "Geriatric Population*" OR "Geriatric Patient*" OR “Aging Population" OR “Aged Population" OR "Aged 60" OR "60 year*" OR "Aged 65" OR "65 year*" OR "Gerodont*" OR "Aged 70" OR "70 year*" OR "Aged 75" OR "75 year*" OR "Aged 80" OR "80 year*" OR “Senior*" OR “Care-depend*" OR "Older Adults" OR “Older People”):ti,ab,kw
2. 'periodontitis'/exp
3. ‘periodont*’:ti,ab,kw
4. 'periodontal index'/exp
5. 2-4/OR
6. 'dental health'/exp
7. 'disease exacerbation'/exp
8. 'edentulous jaw'/exp
9. 'tooth extraction'/exp
10. (‘disease progression’ OR ‘attachment loss’ OR ‘bone loss’ OR ‘periodontal progression’ OR ‘Plaque Index’ OR ‘CPITN’ OR ‘Surviv*’ OR ‘tooth loss’ OR ‘edent*’ OR ‘missing teeth’):ti,ab,kw
11. (tooth NEAR/2 (extract* OR remov*)):ti,ab,kw
12. 'mastication'/exp
13. (‘chew*’ OR ‘masticat*’ OR ‘speech’ OR ‘swallow*’ OR ‘function*’ OR ‘eating’ OR ‘comfort’):ti,ab,kw
14. 'quality of life'/exp
15. (‘oral health-related quality of life’ OR ‘OHRQoL’ OR ‘Oral Health Impact Profile’ OR ‘OHIP’ OR ‘Oral Impact on Daily Performances’ OR ‘OIDP’):ti,ab,kw
16. 6-15/OR
17. 1 OR 4 OR 17

**Results: 2,216**

**Scopus**

1. TITLE-ABS-KEY ( "Elder*" OR "Geriatric Population*" OR "Geriatric Patient*" OR "Aging Population" OR "Aged Population" OR "Aged 60" OR "60 year*" OR "Aged 65" OR "65 year*" OR "Gerodont*" OR "Aged 70" OR "70 year*" OR "Aged 75" OR "75 year*" OR "Aged 80" OR "80 year*" OR "Senior*" OR "Care-depend*" OR "Older Adults" OR "Older People" )
2. Title-ABS-KEY ( "periodont*" )
3. TITLE-ABS-KEY ( "disease progression" OR "attachment loss" OR "bone loss" OR "periodontal progression" OR "CPITN" OR "Surviv*" OR "tooth loss" OR "edent*" OR "missing teeth" OR "chew*" OR "masticat*" OR "speech" OR "swallow*" OR "function*" OR "eating" OR "comfort” OR "oral health-related quality of life" OR "OHRQoL" OR "Oral Health Impact Profile" OR "OHIP" OR "Oral Impact on Daily Performances" OR "OIDP" OR "tooth extract*" OR "tooth remov*" )
4. #1 AND #2 AND #3

**Results: 4,619**

**Cochrane**

1. MeSH descriptor: [Dental Care for Aged] explode all trees
2. (Elder* OR (Geriatric NEXT Population*) OR (Geriatric NEXT Patient*) OR “Aging Population" OR “Aged Population" OR "Aged 60" OR (60 NEXT year*) OR “Aged 65” OR (65 NEXT year*) OR “Aged 70” OR (70 NEXT year*) OR Gerodont* OR "Aged 75" OR (75 NEXT year*) OR "Aged 80" OR (80 NEXT year*) OR Senior* OR Care-depend* OR "Older Adults" OR “Older People”):ti,ab,kw
3. #1 OR #2
4. MeSH descriptor: [Periodontitis] explode all trees
5. periodont*:ti,ab,kw
6. MeSH descriptor: [Periodontal Index] explode all trees
7. #4 OR #5 OR #6
8. MeSH descriptor: [Oral Health] explode all trees
9. MeSH descriptor: [Disease Progression] explode all trees
10. MeSH descriptor: [Tooth Loss] explode all trees
11. MeSH descriptor: [Jaw, Edentulous] explode all trees
12. MeSH descriptor: [Periodontal Atrophy] explode all trees
13. ("disease progression" OR "attachment loss" OR "bone loss" OR "periodontal progression" OR “Plaque Index" OR “CPITN" OR Surviv* OR "tooth loss" OR edent* OR "missing teeth"):ti,ab,kw
14. MeSH descriptor: [Mastication] explode all trees
15. (chew* OR masticat* OR speech OR swallow* OR function* OR eating OR comfort):ti,ab,kw
16. MeSH descriptor: [Quality of Life] explode all trees
17. ("oral health-related quality of life" OR "OHRQoL" OR "Oral Health Impact Profile" OR "OHIP" OR "Oral Impact on Daily Performances" OR "OIDP"):ti,ab,kw
18. MeSH descriptor: [Tooth Extraction] explode all trees
19. (tooth NEAR/2 (extract* OR remov*)):ti,ab,kw
20. #8 OR #9 OR #10 OR #11 OR #12 OR #13 OR #14 OR #15 OR #16 OR #17 OR #18 OR #19
21. #3 AND #7 AND #20

**Review results: 4**

**Trial results: 731**
