# Supplementary material for: The Impact of Periodontitis on Oral Health Outcomes in Older Adults: A Systematic Review
Source: Gerodontology. 2026 Jan 16;43(3):318–38. doi: 10.1111/ger.70048 (PMC13498920; doi:10.1111/ger.70048)
Supplement: Supplementary file 2 [file GER-43-318-s002.docx]

**Supplementary Information S3**

**Table S3a. Risk of Bias Assessment for the Cohort Studies Using the Newcastle–Ottawa Scale**

| Author (year) | Selection | | | | Comparability | | Outcome | | | Total | Quality |
| --- | --- | --- | --- | --- | --- | --- | --- | --- | --- | --- | --- |
|  | S1 | S2 | S3 | S4 | C1 | C2 | R1 | R2 | R3 |  |  |
| Beck et al. 1994 | ★ | ★ | ★ | ☆ | ☆ | ☆ | ★ | ★ | ☆ | 5 | Fair |
| Beck et al. 1997 | ★ | ★ | ★ | ☆ | ☆ | ☆ | ★ | ★ | ☆ | 5 | Fair |
| Beck et al. 1997 | ★ | ★ | ★ | ☆ | ☆ | ☆ | ★ | ★ | ☆ | 5 | Fair |
| Brown et al. 1994 | ★ | ★ | ★ | ☆ | ★ | ☆ | ★ | ★ | ☆ | 6 | Fair |
| Gonda et al. 2013 | ☆ | ★ | ★ | ☆ | ☆ | ☆ | ★ | ★ | ☆ | 4 | Fair |
| Hassel et al. 2018 | ★ | ★ | ★ | ☆ | ★ | ☆ | ★ | ★ | ☆ | 6 | Fair |
| Hirotomi et al. 2002 | ☆ | ★ | ★ | ☆ | ★ | ☆ | ★ | ★ | ☆ | 5 | Fair |
| Hirotomi et al. 2010 | ★ | ★ | ★ | ☆ | ★ | ☆ | ★ | ★ | ☆ | 6 | Fair |
| Hirotomi et al. 2012 | ★ | ★ | ★ | ☆ | ★ | ☆ | ★ | ★ | ☆ | 6 | Fair |
| Iwai et al. 2024 | ☆ | ★ | ★ | ★ | ★ | ★ | ★ | ★ | ★ | 8 | Good |
| Mihara et al. 2020 | ☆ | ★ | ★ | ☆ | ★ | ☆ | ★ | ★ | ☆ | 5 | Fair |
| Nilsson et al. 2019 | ★ | ★ | ★ | ☆ | ☆ | ☆ | ★ | ★ | ☆ | 5 | Fair |
| Nishimoto et al. 2023 | ★ | ★ | ★ | ★ | ★ | ☆ | ★ | ★ | ☆ | 7 | Good |
| Ogawa et al. 2002 | ★ | ★ | ★ | ☆ | ☆ | ☆ | ★ | ★ | ☆ | 5 | Fair |
| Sato et al. (2022) | ☆ | ★ | ★ | ☆ | ★ | ☆ | ★ | ★ | ☆ | 5 | Fair |
| Shimazaki et al. 2003 | ★ | ★ | ★ | ☆ | ★ | ☆ | ★ | ★ | ☆ | 6 | Fair |
| Slade et al. 1997 | ☆ | ★ | ★ | ☆ | ★ | ☆ | ★ | ★ | ☆ | 5 | Fair |
| Warren et al. 2002 | ★ | ★ | ★ | ☆ | ★ | ☆ | ★ | ★ | ☆ | 6 | Fair |
| *Notes.*  S1 representativeness of exposed cohort; S2 selection of non-exposed cohort; S3 ascertainment of exposure; S4 outcome not present at baseline; C1 control for main confounder; C2 control for additional confounders; R1 assessment of outcome; R2 adequacy of follow-up duration; R3 completeness of follow-up / missing data. In the Newcastle–Ottawa Scale, a filled star (★) indicates that the study met the criterion for that item, whereas an empty star (☆) indicates that the criterion was not met or that insufficient information was reported to award a star. | | | | | | | | | | | |

**Table S3b. Risk of Bias Assessment for the Cross-Sectional Studies Using the Modified Newcastle–Ottawa Scale**

| Author (year) | Selection | | | | Comparability | | Outcome | | | Total | Quality |
| --- | --- | --- | --- | --- | --- | --- | --- | --- | --- | --- | --- |
|  | S1 | S2 | S3 | S4 | C1 | C2 | R1 | R2 | R3 |  |  |
| Agustina et al. (2023) | ★ | ☆ | ★ | ★ | ★ | ★ | ☆ | ★ | ☆ | 6 | Fair |
| Botelho et al. (2020) | ★ | ☆ | ☆ | ★ | ★ | ★ | ☆ | ★ | ☆ | 5 | Fair |
| Guevara-Canales et al. (2018) | ☆ | ☆ | ☆ | ★ | ☆ | ☆ | ☆ | ★ | ☆ | 2 | Poor |
| Göktürk & Uçan Yarkaç (2019) | ☆ | ★ | ☆ | ★ | ☆ | ☆ | ☆ | ★ | ☆ | 3 | Poor |
| Marya et al. (2020) | ☆ | ☆ | ☆ | ★ | ☆ | ☆ | ☆ | ★ | ☆ | 2 | Poor |
| León et al. (2016) | ☆ | ☆ | ☆ | ★ | ☆ | ☆ | ☆ | ★ | ☆ | 2 | Poor |
| Kato et al. (2018) | ☆ | ☆ | ☆ | ★ | ★ | ★ | ☆ | ★ | ☆ | 4 | Fair |
| Rekhi et al. (2016) | ☆ | ★ | ☆ | ★ | ☆ | ☆ | ☆ | ★ | ☆ | 3 | Poor |
| Castrejón-Pérez et al. (2016) | ☆ | ☆ | ☆ | ★ | ☆ | ☆ | ☆ | ★ | ☆ | 2 | Poor |
| Hijryana et al. (2022) | ★ | ★ | ★ | ★ | ★ | ★ | ☆ | ★ | ★ | 8 | Good |
| Srisilapanan and Sheiham (2001) | ☆ | ☆ | ☆ | ★ | ★ | ★ | ☆ | ★ | ☆ | 4 | Fair |
| Hunt at al. (1995) | ★ | ☆ | ★ | ★ | ★ | ★ | ☆ | ★ | ☆ | 6 | Fair |
| Swoboda et al. (2006) | ★ | ☆ | ☆ | ★ | ★ | ★ | ☆ | ★ | ☆ | 5 | Fair |
| Masood et al (2017) | ★ | ☆ | ★ | ★ | ★ | ★ | ☆ | ★ | ★ | 7 | Good |
| Sheng et al. (2018) | ★ | ☆ | ☆ | ★ | ★ | ★ | ☆ | ★ | ☆ | 5 | Fair |
| Cornejo et al. (2013) | ☆ | ☆ | ☆ | ★ | ★ | ★ | ☆ | ★ | ☆ | 4 | Fair |
| Marya et al. (2021) | ☆ | ☆ | ☆ | ★ | ★ | ☆ | ☆ | ★ | ☆ | 3 | Poor |
| Shivakumar et al. (2018) | ☆ | ☆ | ☆ | ★ | ★ | ★ | ☆ | ★ | ☆ | 4 | Fair |
| Rani et al. (2024) | ☆ | ☆ | ☆ | ★ | ☆ | ☆ | ☆ | ★ | ☆ | 2 | Poor |
| Göktürk & Uçan Yarkaç, 2018 | ☆ | ★ | ★ | ★ | ☆ | ☆ | ☆ | ★ | ☆ | 4 | Fair |
| Shao et al. (2018) | ★ | ☆ | ☆ | ★ | ★ | ☆ | ☆ | ★ | ☆ | 4 | Fair |
| Sødal et al. (2022) | ★ | ☆ | ☆ | ★ | ★ | ★ | ☆ | ★ | ☆ | 5 | Fair |
| Sukumar et al. (2025) | ☆ | ☆ | ☆ | ★ | ★ | ★ | ☆ | ★ | ☆ | 4 | Fair |
| Zhao et al. (2011) | ★ | ★ | ★ | ★ | ★ | ★ | ☆ | ★ | ★ | 8 | Good |
| Zhi et al. (2022) | ★ | ★ | ☆ | ★ | ★ | ★ | ☆ | ★ | ☆ | 6 | Fair |
| *Notes:*  S1 – representativeness of the sample; S2 – sample size justification; S3 – handling and reporting of non-respondents; S4 – ascertainment of exposure (validated measurement); C1 – comparability via adjustment for the most important confounder; C2 – comparability via adjustment for additional confounders; R1 – assessment of outcome (independent vs. self-report); R2 – appropriateness and clarity of statistical testing; R3 – reporting and handling of missing data. In the modified Newcastle–Ottawa Scale, a filled star (★) indicates that the study met the criterion for that item, whereas an empty star (☆) indicates that the criterion was not met or that insufficient information was reported to award a star. | | | | | | | | | | | |
