# Supplementary material for: The Impact of Periodontitis on Oral Health Outcomes in Older Adults: A Systematic Review
Source: Gerodontology. 2026 Jan 16;43(3):318–38. doi: 10.1111/ger.70048 (PMC13498920; doi:10.1111/ger.70048)
Supplement: Supplementary file 3 [file GER-43-318-s003.docx]

**Supplementary information S2**

Table listing studies that were excluded following full text review (n=84)

| **No.** | **Author (year)** | **Study title** | **Reason for exclusion** |
| --- | --- | --- | --- |
| 1 | Ajwani (2001) | Periodontal conditions among the old elderly: five-year longitudinal study | Wrong outcomes |
| 2 | Al-Harthi (2022) | Tooth loss and radiographic bone loss in patients without regular supportive care: A retrospective study | Age breakdown for ≥60 not given |
| 3 | Al-Kattan (2019) | Influence of Periodontal Disease on QoL of Periodontal Patients in Riyadh | Age breakdown for ≥60 not given |
| 4 | Al Mosawi (2021) | Need and Demand According to Basic Periodontal Index to Patient with Periodontal Disease Attending Department of Periodontology at the College of Dentistry/University of Baghdad | Wrong study design |
| 5 | Andrade (2019) | Oral health profile among community-dwelling elderly and its association with self-rated oral health | No OHQOL by periodontal status analysis carried out |
| 6 | Angst (2024) | Oral health and nutritional status in care-dependent, community-dwelling older adults in Zurich, Switzerland | No OHQOL by periodontal status analysis carried out |
| 7 | Ansari Moghadam (2015) | The Relationship Between Periodontal Disease and Public Health: A Population-Based Study | Age breakdown for ≥60 not given |
| 8 | Appukuttan (2016) | Oral Health Related Quality of Life among Tamil Speaking Adults Attending a Dental Institution in Chennai, Southern India | Age breakdown for ≥60 not given |
| 9 | Åslund (2008) | Measuring oral health-related quality-of-life using OHQoL-GE in periodontal patients presenting at the University of Berne, Switzerland | Age breakdown for ≥60 not given |
| 10 | Axelsson (2004) | The long-term effect of a plaque control program on tooth mortality, caries and periodontal disease in adults. Results after 30 years of maintenance | Age breakdown for ≥60 not given |
| 11 | Baelum (1997) | Predictors of tooth loss over 10 years in adult and elderly Chinese | Age breakdown for ≥60 not given |
| 12 | Baelum (1997) | Predictors of destructive periodontal disease incidence and progression in adult and elderly Chinese | Age breakdown for ≥60 not given |
| 13 | Baelum (1997) | A 10-year study of the progression of destructive periodontal disease in adult and elderly Chinese | No outcome by periodontal status analysis carried out |
| 14 | Baelum (1993) | Six-year progression of destructive periodontal disease in 2 subgroups of elderly Chinese | Age breakdown for ≥60 not given |
| 15 | Baelum (1988) | Tooth mortality and periodontal conditions in 60–80-year-old Chinese | Wrong study design |
| 16 | Beck (1997) | A 5-year study of attachment loss in community-dwelling older adults: incidence density | No outcome by periodontal status analysis carried out |
| 17 | Beck (1994) | Characteristics of older adults experiencing periodontal attachment loss as gingival recession or probing depth | Wrong outcomes |
| 18 | Beck (1995) | Incidence of attachment loss over 3 years in older adults—new and progressing lesions | No outcome by periodontal status analysis carried out |
| 19 | Bernabé (2010) | Periodontal disease and quality of life in British adults | Age breakdown for ≥60 not given |
| 20 | Bianco (2021) | Oral health status and the impact on oral health-related quality of life among the institutionalized elderly population: a cross-sectional study in an area of southern Italy | Wrong outcomes |
| 21 | Bianco (2010) | The impact on life quality due to oral conditions in people fifty years or above | Age breakdown for ≥60 not given |
| 22 | Buchwald (2013) | Tooth loss and periodontitis by socio-economic status and inflammation in a longitudinal population-based study | Age breakdown for ≥60 not given |
| 23 | Burt (1985) | Periodontal disease, tooth loss, and oral hygiene among older Americans | Wrong study design |
| 24 | Canga (2022) | THE IMPACT OF THE DIABETES ON ORAL HEALTH - AN OBSERVATIONAL STUDY | Wrong study design |
| 25 | Chairunisa (2024) | Ten years' evaluation of periodontal status and its changes among Japanese older adults | No outcome by periodontal status analysis carried out |
| 26 | Chambrone (2006) | Tooth loss in well-maintained patients with chronic periodontitis during long-term supportive therapy in Brazil | No outcome by periodontal status analysis carried out |
| 27 | Chatrchaiwiwatana (2007) | Factors affecting tooth loss among rural Khon Kaen adults: Analysis of two data sets | Wrong study design |
| 28 | Chengrui (2025) | The symptom network of oral health conditions in older populations with oral frailty: a cross-sectional study | No OHQOL by periodontal status analysis carried out |
| 29 | Cunha-Cruz (2007) | Oral health-related quality of life of periodontal patients | Age breakdown for ≥60 not given |
| 30 | Dahlén (1995) | Periodontopathogens in elderly Chinese with different periodontal disease experience | Age breakdown for ≥60 not given |
| 31 | de Andrade (2018) | Oral health profile among community-dwelling elderly and its association with self-rated oral health | Wrong outcomes |
| 32 | De Marchi (2012) | Four-year incidence and predictors of tooth loss among older adults in a southern Brazilian city | Periodontal diagnosis not clear |
| 33 | Drake (1995) | Three-year tooth loss among black and white older adults in North Carolina | Periodontal diagnosis not clear |
| 34 | Edman (2022) | Periodontal health and disease in an older population: A 10-year longitudinal study | No outcome by periodontal status analysis carried out |
| 35 | Elter (1999) | Etiologic models for incident periodontal attachment loss in older adults | No outcome by periodontal status analysis carried out |
| 36 | Enoki (2013) | Determinants of change in oral health-related quality of life over 7 years among older Japanese | No OHQOL by periodontal status analysis carried out |
| 37 | Hijryana (2022) | Impact of Periodontal Disease on the Quality of Life of Older People in Indonesia: A Qualitative Study | Wrong study design |
| 38 | Hiltunen (2023) | Why and when older people lose their teeth: A study of public healthcare patients aged 60 years and over in 2007–2015 | No outcome by periodontal status analysis carried out |
| 39 | Hoeksema (2018) | Health and quality of life differ between community living older people with and without remaining teeth who recently received formal home care: a cross-sectional study | No OHQOL by periodontal status analysis carried out |
| 40 | Hugoson (1995) | Oral health of individuals aged 3–80 years in Jönköping, Sweden in 1973, 1983, and 1993. II. Review of clinical and radiographic findings | No outcome by periodontal status analysis carried out |
| 41 | Jansson (2014) | Impact of periodontal disease experience on oral health-related quality of life | Age breakdown for ≥60 not given |
| 42 | Jansson (2002) | Marginal bone loss and tooth loss in a sample from the County of Stockholm—a longitudinal study over 20 years | Age breakdown for ≥60 not given |
| 43 | Joshi (1996) | Consequences of success: do more teeth translate into more disease and utilization? | Wrong study design |
| 44 | Ju (2022) | A Longitudinal Study of Chronic Periodontitis in Two Cohorts of Community-Dwelling Elderly Australians | No outcome by periodontal status analysis carried out |
| 45 | Kosaka (2016) | A multifactorial model of masticatory performance: the Suita study | Wrong study design |
| 46 | Kumar (2024) | The association between denture care and oral hygiene habits, oral hygiene knowledge and periodontal status of geriatric patients wearing removable partial dentures | Wrong study design |
| 47 | Lee (2022) | Oral health status and behavior in elderly Koreans with periodontal disease | Wrong study design |
| 48 | Liang (2020) | Impact of periodontal disease and chewing ability on the quality of life of the elderly in an affluent community | Periodontal diagnosis not clear |
| 49 | Locker (1997) | Clinical correlates of changes in self-perceived oral health in older adults | Wrong outcomes |
| 50 | Locker (1996) | Incidence of and risk factors for tooth loss in a population of older Canadians | Age breakdown for ≥60 not given |
| 51 | Mariño (2008) | Self-reported and clinically determined oral health status predictors for quality of life in dentate older migrant adults | Age breakdown for ≥60 not given |
| 52 | McGrath (2002) | Oral health behind bars: a study of oral disease and its impact on the life quality of an older prison population | No OHQOL by periodontal status analysis carried out |
| 53 | Meneses-Gómez (2016) | Oral health-related quality of life in the elderly population receiving health care at the public hospital network in Medellín, Colombia, and its related factors | No OHQOL by periodontal status analysis carried out |
| 54 | Moriya (2012) | Factors associated with self-assessed masticatory ability among community-dwelling elderly Japanese | Wrong study design |
| 55 | Motallebnejad (2015) | The evaluation of oral health-related factors on the quality of life of the elderly in Babol | No OHQOL by periodontal status analysis carried out |
| 56 | M (2021) | Critical Assessment on Unmet Oral Health Needs and Oral Health-related Quality of Life Among Old Age Home Inhabitants in Karnataka, India | No OHQOL by periodontal status analysis carried out |
| 57 | Natto (2014) | Factors contributing to tooth loss among the elderly: A cross sectional study | Wrong study design |
| 58 | Neelamana (2020) | Oral health status and related quality of life among elderly tribes in India | No OHQOL by periodontal status analysis carried out |
| 59 | Nordström (1998) | A 9-year longitudinal study of reported oral problems and dental and periodontal status in 70- and 79-year-old city cohorts in northern Sweden | No outcome by periodontal status analysis carried out |
| 60 | Okada (2014) | Association of periodontal status with occlusal force and food acceptability in 70-year-old adults: from SONIC Study | Wrong study design |
| 61 | Papapanou (1989) | A 10-year retrospective study of periodontal disease progression | No outcome by periodontal status analysis carried out |
| 62 | Petrović (2017) | Oral health-related quality of life of institutionalized elderly in Serbia | No OHQOL by periodontal status analysis carried out |
| 63 | Qian (2017) | Self-reported oral hygiene care and natural teeth retention among older Singaporeans | Periodontal diagnosis not clear |
| 64 | Ramsay (2015) | Burden of poor oral health in older age: findings from a population-based study of older British men | No OHQOL by periodontal status analysis carried out |
| 65 | Ramseier (2017) | Natural history of periodontitis: Disease progression and tooth loss over 40 years | Age breakdown for ≥60 not given |
| 66 | Ravald (2012) | Tooth loss in periodontally treated patients. A long-term study of periodontal disease and root caries | Age breakdown for ≥60 not given |
| 67 | Ravi (2019) | Relationship between geriatric oral health assessment index (GOHAI) and oral health status of the institutionalized elderly in Mangalore, India | No OHQOL by periodontal status analysis carried out |
| 68 | Rosli (2017) | H6: Association between oral health conditions, oral health-related quality of life and nutritional status among older adults | Periodontal diagnosis not clear |
| 69 | Sabbah (2011) | The relationship between social network, social support and periodontal disease among older Americans | No OHQOL by periodontal status analysis carried out |
| 70 | Schmalz (2021) | Oral Health-Related Quality of Life, Oral Conditions, and Risk of Malnutrition in Older German People in Need of Care – A Cross-Sectional Study | No OHQOL by periodontal status analysis carried out |
| 71 | Sharma (2022) | A Cross-Sectional Epidemiological Study To Assess The Dental Caries Status, Periodontal Status And Its Association With OHRQoL Among The Elderly Residents Of Aged Care Homes In Punjab | No archived study available |
| 72 | Sha (2019) | Assessment of periodontal health and necessity of dental treatment in the institutionalized elderly population of East Godavari district, Andhra Pradesh | Wrong study design |
| 73 | Sheiham (2001) | Prevalence of impacts of dental and oral disorders and their effects on eating among older people; a national survey in Great Britain | Periodontal diagnosis not clear |
| 74 | Shetty (2013) | The relationship between prosthetic status and the Geriatric Oral Health Assessment Index in a group of institutionalized elderly of an Indian city: a cross-sectional study | Periodontal diagnosis not clear |
| 75 | Sonnenschein (2018) | Oral health-related quality of life in patients under supportive periodontal therapy | Age breakdown for ≥60 not given |
| 76 | Stamadianos (2009) | Oral health and quality of life of dental patients in the General Hospital of Kalymnos | Age breakdown for ≥60 not given |
| 77 | Thorstensson (2010) | Why do some people lose teeth across their lifespan whereas others retain a functional dentition into very old age? | No outcome by periodontal status analysis carried out |
| 78 | Tôrres (2023) | Predictors of tooth loss in Brazilian older adults: An 8-year follow-up | Periodontal diagnosis not clear |
| 79 | Ueno (2022) | Periodontal status is associated with oral function in community-dwelling older adults, independent of dentition status | Wrong study design |
| 80 | Vaziri (2016) | Oral Health-Related Quality of Life and Severity of Periodontal Disease | Age breakdown for ≥60 not given |
| 81 | Wennström (1993) | Periodontal conditions of adult regular dental care attendants. A 12-year longitudinal study | Age breakdown for ≥60 not given |
| 82 | Wu (2001) | A 10-year longitudinal study of the progression of destructive periodontal disease in adult and elderly | Age breakdown for ≥60 not given |
| 83 | Zeng (2010) | Relationship between dental status and Oral Impacts on Daily Performances in older Southern Chinese people | Age breakdown for ≥60 not given |
| 84 | Zhao (2024) | Associations of Social Psychological Factors and OHRQoL in Periodontitis Patients: A Structural Equation Modeling Study | No OHQOL by periodontal status analysis carried out |

**References**

1. Ajwani S, Ainamo A. Periodontal conditions among the old elderly: five-year longitudinal study. Spec Care Dentist. 2001;21(2):45–51.
2. Al-Harthi S, Barbagallo G, Psaila A, d’Urso U, Nibali L. Tooth loss and radiographic bone loss in patients without regular supportive care: a retrospective study. J Periodontol. 2022;93(3):354–63.
3. Al-Kattan R, Shibani NA, Zahrani HAL, Enazi GA, Nafa DA. Influence of periodontal disease on quality of life of periodontal patients in Riyadh. J Adv Oral Res. 2019;10(2):85–90.
4. Al Mosawi AO. Need and demand according to basic periodontal index to patient with periodontal disease attending the Department of Periodontology, College of Dentistry, University of Baghdad. Lat Am J Pharm. 2021;40(Spec Iss):271–5.
5. Andrade FB, Teixeira DSC, Frazão P, Duarte YAO, Lebrão ML, Antunes JLF. Oral health profile among community-dwelling elderly and its association with self-rated oral health. Rev Bras Epidemiol. 2019;21(Suppl 2):e180012.
6. Angst L, Ferreira Lourenço PD, Srinivasan M. Oral health and nutritional status in care-dependent, community-dwelling older adults in Zurich, Switzerland. Swiss Dent J. 2024;134(2):122–44.
7. Ansari Moghadam S, Abdollahi Z, Risbaf Fakour S, Ansari Moghaddam A, Kiany F, Damani N. The relationship between periodontal disease and public health: a population-based study. Glob J Health Sci. 2015;8(7):110–5.
8. Appukuttan DP, Tadepalli A, Victor DJ, Dharuman S. Oral health-related quality of life among Tamil-speaking adults attending a dental institution in Chennai, Southern India. J Clin Diagn Res. 2016;10(10):ZC114–20.
9. Åslund M, Pjetursson BE, Lang NP. Measuring oral health-related quality of life using OHQoL-GE in periodontal patients. Oral Health Prev Dent. 2008;6(3):191–7.
10. Axelsson P, Nyström B, Lindhe J. The long-term effect of a plaque control program on tooth mortality, caries and periodontal disease in adults: results after 30 years of maintenance. J Clin Periodontol. 2004;31(9):749–57.
11. Baelum V, Luan WM, Chen X, Fejerskov O. Predictors of tooth loss over 10 years in adult and elderly Chinese. Community Dent Oral Epidemiol. 1997;25(3):204–10.
12. Baelum V, Luan WM, Chen X, Fejerskov O. Predictors of destructive periodontal disease incidence and progression in adult and elderly Chinese. Community Dent Oral Epidemiol. 1997;25(4):265–72.
13. Baelum V, Luan WM, Chen X, Fejerskov O. A 10-year study of the progression of destructive periodontal disease in adult and elderly Chinese. J Periodontol. 1997;68(11):1033–42.
14. Baelum V, Wen-Min L, Dahlen G, Fejerskov O, Xia C. Six-year progression of destructive periodontal disease in two subgroups of elderly Chinese. J Periodontol. 1993;64(9):891–9.
15. Baelum V, Wen-Min L, Fejerskov O, Xia C. Tooth mortality and periodontal conditions in 60–80-year-old Chinese. Scand J Dent Res. 1988;96(2):99–107.
16. Beck JD, Cusmano L, Green-Helms W, Koch GG, Offenbacher S. A 5-year study of attachment loss in community-dwelling older adults: incidence density. J Periodontal Res. 1997;32(6):506–15.
17. Beck JD, Koch GG. Characteristics of older adults experiencing periodontal attachment loss as gingival recession or probing depth. J Periodontal Res. 1994;29(4):290–8.
18. Beck JD, Koch GG, Offenbacher S. Incidence of attachment loss over 3 years in older adults—new and progressing lesions. Community Dent Oral Epidemiol. 1995;23(5):291–6.
19. Bernabé E, Marcenes W. Periodontal disease and quality of life in British adults. J Clin Periodontol. 2010;37(11):968–72.
20. Bianco A, Mazzea S, Fortunato L, Giudice A, Papadopoli R, Nobile CGA, et al. Oral health status and its impact on oral health-related quality of life among institutionalized elderly. Int J Environ Res Public Health. 2021;18(4):2175.
21. Bianco VC, Lopes ES, Borgato MH, Silva PM, Marta SN. Impact on quality of life due to oral conditions in people aged 50 years or above. Cien Saude Colet. 2010;15(4):2165–72.
22. Buchwald S, Kocher T, Biffar R, Harb A, Holtfreter B, Meisel P. Tooth loss and periodontitis by socio-economic status and inflammation. J Clin Periodontol. 2013;40(3):203–11.
23. Burt BA, Ismail AI, Eklund SA. Periodontal disease, tooth loss, and oral hygiene among older Americans. Community Dent Oral Epidemiol. 1985;13(2):93–6.
24. Canga M, Malagnino I, Qafmolla A, Xhajanka E, Malagnino VA. Impact of diabetes on oral health. Wiad Lek. 2022;75(4):753–8.
25. Chairunisa F, Widita E, Thwin KM, Takehara S, Nohno K, Hanindriyo L, et al. Ten-year evaluation of periodontal status among Japanese older adults. Spec Care Dentist. 2024;44(6):1731–41.
26. Chambrone LA, Chambrone L. Tooth loss in well-maintained patients with chronic periodontitis. J Clin Periodontol. 2006;33(10):759–64.
27. Chatrchaiwiwatana S. Factors affecting tooth loss among rural Thai adults. Public Health. 2007;121(2):106–12.
28. Chengrui Z, Ying X, Wei L, Bin C. Symptom network of oral health conditions in older populations with oral frailty. BMC Oral Health. 2025;25:471.
29. Cunha-Cruz J, Hujoel PP, Kressin NR. Oral health-related quality of life of periodontal patients. J Periodontal Res. 2007;42(2):169–76.
30. Dahlén G, Luan WM, Baelum V, Fejerskov O, Chen X. Periodontopathogens in elderly Chinese. J Clin Periodontol. 1995;22(3):188–200.
31. de Andrade FB, Teixeira DSC, Frazão P, Duarte YAO, Lebrão ML, Antunes JLF. Oral health profile among community-dwelling elderly. Rev Bras Epidemiol. 2018;21:e180012.
32. De Marchi RJ, Hilgert JB, Hugo FN, Santos CMD, Martins AB, Padilha DM. Four-year incidence and predictors of tooth loss. Community Dent Oral Epidemiol. 2012;40(5):396–405.
33. Drake CW, Hunt RJ, Koch GG. Three-year tooth loss among black and white older adults. J Dent Res. 1995;74(2):675–80.
34. Edman K, Norderyd O, Holmlund A. Periodontal health and disease in an older population: a 10-year study. Community Dent Oral Epidemiol. 2022;50(4):225–32.
35. Elter JR, Beck JD, Slade GD, Offenbacher S. Etiologic models for incident periodontal attachment loss. J Clin Periodontol. 1999;26(2):113–23.
36. Enoki K, Ikebe K, Matsuda KI, Yoshida M, Maeda Y, Thomson WM. Determinants of change in oral health-related quality of life over 7 years. J Oral Rehabil. 2013;40(4):252–7.
37. Hijryana M, MacDougall M, Ariani N, Kusdhany LS, Walls AWG. Impact of periodontal disease on quality of life of older people. JDR Clin Trans Res. 2022;7(4):360–70.
38. Hiltunen K, Vehkalahti MM. Why and when older people lose their teeth. Gerodontology. 2023;40(3):326–33.
39. Hoeksema AR, Peters LL, Raghoebar GM, Meijer HJA, Vissink A, Visser A. Health and quality of life in older people with and without remaining teeth. Clin Oral Investig. 2018;22(7):2615–22.
40. Hugoson A, Koch G, Bergendal T, Hallonsten AL, Slotte C, Thorstensson B, et al. Oral health in individuals aged 3–80 years in Jönköping, Sweden. Swed Dent J. 1995;19(6):243–60.
41. Jansson H, Wahlin A, Johansson V, Åkerman S, Lundegren N, Isberg PE, et al. Impact of periodontal disease experience on oral health-related quality of life. J Periodontol. 2014;85(3):438–45.
42. Jansson L, Lavstedt S, Zimmerman M. Marginal bone loss and tooth loss over 20 years. Swed Dent J. 2002;26(1):21–9.
43. Joshi A, Douglass CW, Feldman H, Mitchell P, Jette A. Consequences of success: do more teeth translate into more disease? J Public Health Dent. 1996;56(4):190–7.
44. Ju X, Harford J, Luzzi L, Mejia G, Jamieson LM. Longitudinal study of chronic periodontitis in elderly Australians. Int J Environ Res Public Health. 2022;19(18):11824.
45. Kosaka T, Ono T, Kida M, Kikui M, Yamamoto M, Yasui S, et al. Multifactorial model of masticatory performance. J Oral Rehabil. 2016;43(5):340–7.
46. Kumar A. Denture care, oral hygiene habits and periodontal status of geriatric patients. Int J Life Sci Biotechnol Pharm Res. 2024;13(7):166–9.
47. Lee SR, Han MA, Park J, Ryu SY, Kim SY. Oral health status in elderly Koreans with periodontal disease. J Public Health Dent. 2022;82(4):378–84.
48. Liang YH, Chou C, Chen YJ, Chou YF, Lin CY, Wang TF. Periodontal disease, chewing ability and quality of life. J Formos Med Assoc. 2020;119(11):1693–701.
49. Locker D. Clinical correlates of changes in self-perceived oral health. Community Dent Oral Epidemiol. 1997;25(3):199–203.
50. Locker D, Ford J, Leake JL. Incidence of and risk factors for tooth loss. J Dent Res. 1996;75(2):783–9.
51. Mariño R, Schofield M, Wright C, Calache H, Minichiello V. Oral health status predictors for quality of life. Community Dent Oral Epidemiol. 2008;36(1):85–94.
52. McGrath C. Oral health behind bars. Gerodontology. 2002;19(2):109–14.
53. Meneses-Gómez EJ, Posada-López A, Agudelo-Suarez AA. Oral health-related quality of life in elderly Colombians. Acta Odontol Latinoam. 2016;29(2):151–61.
54. Moriya S, Tei K, Muramatsu T, Murata A, Muramatsu M, Harada E, et al. Self-assessed masticatory ability. Community Dent Health. 2012;29(1):39–44.
55. Motallebnejad M, Mehdizadeh S, Najafi N, Sayyadi F. Oral health-related quality of life in elderly Iranians. Contemp Clin Dent. 2015;6(3):313–7.
56. M R, Sen M, Mala K, Sujir N, Poojary D, Shetty NJ, et al. Unmet oral health needs in old-age homes. Clin Cosmet Investig Dent. 2021;13:181–6.
57. Natto ZS, Aladmawy M, Alasqah M, Papas A. Factors contributing to tooth loss among the elderly. Singapore Dent J. 2014;35:17–22.
58. Neelamana SK, Janakiram C, Varma B. Oral health status and quality of life among elderly tribes. J Fam Med Prim Care. 2020;9(12):5976–81.
59. Nordström G, Bergman B, Borg K, Nilsson H, Tillberg A, Wenslöv JH. Longitudinal study of oral problems in older adults. Acta Odontol Scand. 1998;56(2):76–84.
60. Okada T, Ikebe K, Inomata C, Takeshita H, Uota M, Mihara Y, et al. Periodontal status and occlusal force. J Oral Rehabil. 2014;41(12):912–9.
61. Papapanou PN, Wennström JL, Gröndahl K. Ten-year retrospective study of periodontal disease progression. J Clin Periodontol. 1989;16(7):403–11.
62. Petrović M, Stančić I, Popovac A, Vasović M. Oral health-related quality of life of institutionalized elderly. Vojnosanit Pregl. 2017;74(5):402–9.
63. Qian Y, Wu B, Sim C. Oral hygiene care and tooth retention among older Singaporeans. Ann Glob Health. 2017;83(1):177.
64. Ramsay SE, Whincup PH, Watt RG, Tsakos G, Papacosta AO, Lennon LT, et al. Burden of poor oral health in older age. BMJ Open. 2015;5(12):e009476.
65. Ramseier CA, Anerud A, Dulac M, Lulic M, Cullinan MP, Seymour GJ, et al. Natural history of periodontitis over 40 years. J Clin Periodontol. 2017;44(12):1182–91.
66. Ravald N, Johansson CS. Tooth loss in periodontally treated patients. J Clin Periodontol. 2012;39(1):73–9.
67. Ravi P, Rao A, Rajesh G, Shenoy R, Pai BHM. GOHAI and oral health status in institutionalized elderly. Indian J Public Health Res Dev. 2019;10(4):146–51.
68. Rosli TI, Kadir RA, Chan YM, Hamid TA. Oral health, OHRQoL and nutritional status among older adults. Med J Malaysia. 2017;72:9.
69. Sabbah W, Tsakos G, Chandola T, Newton T, Kawachi I, Sheiham A, et al. Social support and periodontal disease. J Clin Periodontol. 2011;38(6):547–52.
70. Schmalz G, Denkler CR, Kottmann T, Rinke S, Ziebolz D. Oral health-related quality of life and malnutrition risk. J Clin Med. 2021;10(3):426.
71. Sharma M, Mahajan A, Surendran V, Abhinav, Singh S, Kaur S, et al. Periodontal status and OHRQoL in aged care homes. J Cardiovasc Dis Res. 2022;13(8):3461–70.
72. Sha S, Khan A, Eswara K, Venkata Suvarna D, Kannaiyan K, Pottem N. Periodontal health in institutionalized elderly. J Pharm Bioallied Sci. 2019;11(6):S188–93.
73. Sheiham A, Steele JG, Marcenes W, Tsakos G, Finch S, Walls AWG. Oral impacts on eating among older people. Community Dent Oral Epidemiol. 2001;29(3):195–203.
74. Shetty VD, Bijle MN, Patil S. Prosthetic status and GOHAI. J Contemp Dent Pract. 2013;14(6):1173–7.
75. Sonnenschein SK, Betzler C, Kohnen R, Krisam J, Kim TS. Oral health-related quality of life under supportive periodontal therapy. Acta Odontol Scand. 2018;76(8):572–9.
76. Stamadianos B, Alexias G, Anagnostopoulos F. Oral health and quality of life in hospital dental patients. Arch Hell Med. 2009;26(4):503–16.
77. Thorstensson H, Johansson B. Tooth loss across the lifespan. Gerodontology. 2010;27(1):19–25.
78. Tôrres LHDN, Hilgert JB, Hugo FN, Sousa MDLR, De Marchi RJ. Predictors of tooth loss in Brazilian older adults. Gerodontology. 2023;40(2):207–12.
79. Ueno Y, Iwasaki M, Kimura Y, Kakuta S, Masaki C, Wada T, et al. Periodontal status and oral function. J Periodontal Res. 2022;57(6):1139–47.
80. Vaziri F, Haerian A, Morowati M, Amirian E, Gholamin P. Oral health-related quality of life and periodontal severity. J Int Oral Health. 2016;8(4):440–4.
81. Wennström JL, Serino G, Lindhe J, Eneroth L, Tollskog G. Periodontal conditions of adult regular dental care attendants. J Clin Periodontol. 1993;20(10):714–22.
82. Wu X, Jiang Y, Guo Z. Ten-year longitudinal study of destructive periodontal disease. Zhonghua Kou Qiang Yi Xue Za Zhi. 2001;36(2):108–11.
83. Zeng X, Sheiham A, Bernabé E, Tsakos G. Dental status and oral impacts on daily performances. J Public Health Dent. 2010;70(2):101–7.
84. Zhao C, Wang D, Zhang J, Ge S, Zhan Z, Xu L, et al. Social psychological factors and oral health-related quality of life in periodontitis patients. Patient Prefer Adherence. 2024;18:2359–72.
